# OncoNEM: inferring tumor evolution from single-cell sequencing data

**DOI:** 10.1186/s13059-016-0929-9

**Published:** 2016-04-15

**Authors:** Edith M. Ross, Florian Markowetz

**Affiliations:** Cancer Research UK Cambridge Institute, University of Cambridge, Robinson Way, Cambridge, UK

**Keywords:** Tumor evolution, Cancer evolution, Tumor heterogeneity, Single-cell sequencing, Phylogenetic tree

## Abstract

Single-cell sequencing promises a high-resolution view of genetic heterogeneity and clonal evolution in cancer. However, methods to infer tumor evolution from single-cell sequencing data lag behind methods developed for bulk-sequencing data. Here, we present OncoNEM, a probabilistic method for inferring intra-tumor evolutionary lineage trees from somatic single nucleotide variants of single cells. OncoNEM identifies homogeneous cellular subpopulations and infers their genotypes as well as a tree describing their evolutionary relationships. In simulation studies, we assess OncoNEM’s robustness and benchmark its performance against competing methods. Finally, we show its applicability in case studies of muscle-invasive bladder cancer and essential thrombocythemia.

**Electronic supplementary material:**

The online version of this article (doi:10.1186/s13059-016-0929-9) contains supplementary material, which is available to authorized users.

## Background

Tumor development has long been recognized as an evolutionary process during which a cell population accumulates mutations over time and evolves into a mix of genetically distinct cell subpopulations, called clones [1]. The genetic intra-tumor heterogeneity that develops during clonal evolution poses a major challenge to cancer therapy, as it increases the chance of drug resistance and therefore treatment failure and relapse. Reliable methods for the inference of tumor life histories are important for cancer research, as they provide insights into earlier stages of cancer development and allow predictions about clinical outcome [2]. Furthermore, tumor life histories facilitate the discovery of mutations driving growth and resistance development, as well as the identification of unifying patterns of cancer evolution [3], thereby providing an important stepping-stone towards enhanced treatment strategies for cancer. Inferring the evolutionary history of a tumor, however, remains challenging. Most methods developed for the inference of tumor evolution use data derived from bulk-sequencing of tumor samples, e.g., [4–6]. This approach requires deconvolution of the mixed signal of different tumor subpopulations, which is often ambiguous [7].

### Challenges in single-cell sequencing

Recent advances in single-cell sequencing technologies have promised to reveal tumor heterogeneity at a much higher resolution [8–10]. However, single-cell sequencing comes with its own challenges.

The first challenge is noise in the observed genotypes, which includes false positive and false negative mutations as well as missing values. Reported false discovery rates vary from 2.67×10^−5^ to 6.7×10^−5^ [9–11], which means that false positives can easily outnumber true somatic variants [12]. The number of false positives is usually reduced by census-based variant calling, which only selects variants that are observed in multiple cells, but cannot remove sites of recurrent sequencing errors [13]. Reported allele dropout (ADO) rates vary from 0.16 to 0.43, yielding single nucleotide variant (SNV) data sets with large fractions of false negatives [9–11]. Related to this are missing values, which occur if all copies of a genetic locus fail to amplify, a very common problem in single-cell sequencing data sets [9–11]. Due to this noise, standard clustering methods often fail to identify subpopulations among the sequenced cells, turning even a seemingly simple task, such as mapping cells to clones, into a challenge.

The second challenge lies in unobserved subpopulations. Due to sampling biases, undersampling or extinction of subpopulations, the sampled cells are likely to represent only a subset of the subpopulations that evolved during the tumor’s life history. Thus, methods need to be able to infer unobserved ancestral subpopulations to retrace the evolution of a tumor accurately.

### OncoNEM

Here, we describe OncoNEM (oncogenetic nested effects model), an automated method for reconstructing clonal lineage trees from somatic single nucleotide variants (SSNVs) of multiple single tumor cells that exploits the nested structure of mutation patterns of related cells.

OncoNEM probabilistically accounts for genotyping errors and tests for unobserved subpopulations, addressing both of the challenges described above. It simultaneously clusters cells with similar mutation patterns into subpopulations and infers relationships and genotypes of observed and unobserved subpopulations, yielding results that are more accurate than those of previous methods.

### Existing methods

To gain insights into the evolutionary histories of tumors, various methods have been applied to single-cell data sets of somatic SNVs. Many studies use classic phylogenetic approaches. Examples include UPGMA used by Yu et al. [14] and neighbor joining used by Xu et al. [9], which are both closely related to hierarchical clustering. Hughes et al. [15] used neighbor joining trees as input for a likelihood optimization method, which is based on a general time-reversible substitution model. Another classic phylogenetic approach is Bayesian phylogenetic inference as used by Eirew et al. [16]. None of these methods model the noise of single-cell data sets or infer trees based on subpopulations of cells.

Other studies use non-traditional methods. Some methods first cluster cells into subpopulations and then infer minimum spanning trees. Gawad et al. [17] do this using model-based clustering, whereas Yuan et al. [18] use k-means and hierarchical clustering. Another method is BitPhylogeny, which uses a tree structured mixture model [18]. While mixture models are widely used and valuable, e.g., for inferring the clonal composition of bulk-sequenced samples [5, 6], they require large data sets in order to converge to an accurate representation of the underlying distributions. Current single-cell data sets in contrast are small, containing usually fewer than 100 cells [8–12, 14, 15, 19]. Kim and Simon [20] proposed a method for inferring mutation trees. These are trees in which each node corresponds to a mutation instead of a clone.

For completeness, we also mention approaches that are not applicable in our case, because they are not fully automated or use other types of single-cell data. Li et al. [11] and Melchor et al. [21] performed partially manual inference. Potter et al. [22] defined subpopulations by grouping cells with identical genotypes into clones and then applied a maximum parsimony approach. Their data sets were derived by single-cell qPCR of a few genetic markers, whereas our study focuses on noisy single-cell data sets with hundreds of genetic markers. In these large data sets, the observed genotypes differ between any two cells and the method used by Potter et al. [22] is therefore not applicable. Like some of the studies mentioned above, Navin et al. [8] and Wang et al. [19] used neighbor joining but applied it to single-cell copy-number profiles obtained by whole-genome sequencing. Chowdhury et al. [2, 23] used Steiner trees to infer phylogenies from single-cell copy number profiles obtained from fluorescent in situ hybridization. Their algorithms, however, only infer trees from low-dimensional genotype spaces.

### Outline

In the following, we first explain how OncoNEM infers clonal lineage trees from noisy SSNVs of single cells. Then we assess the robustness of OncoNEM and compare its performance with that of competing methods, which were chosen to be a representative selection of the approaches mentioned above. Finally, we describe the results of applying OncoNEM in two case studies: a data set containing 44 single tumor cells from a muscle-invasive bladder transitional cell carcinoma and a data set containing 58 single tumor cells from an essential thrombocythemia.

## Results and discussion

### Inferring clonal evolution with OncoNEM

The inputs to OncoNEM are (1) a binary genotype matrix containing the observed genotypes of every cell at every SSNV locus and (2) the false positive rate (FPR) *α* and false negative rate (FNR) *β*, which can be estimated from data (see ‘Materials and methods’).

The OncoNEM output includes (1) inferred tumor subpopulations, (2) a tree describing evolutionary relationships between these subpopulations and (3) posterior probabilities of the occurrence of mutations.

The OncoNEM algorithm consists of two main parts: (1) a probabilistic score that models the accumulation of mutations by noisy subset relations and (2) a sequence of inference algorithms to search for high-scoring models in the space of possible tree structures.

#### Probabilistic score for accumulation of mutations

**Fig. 1:**
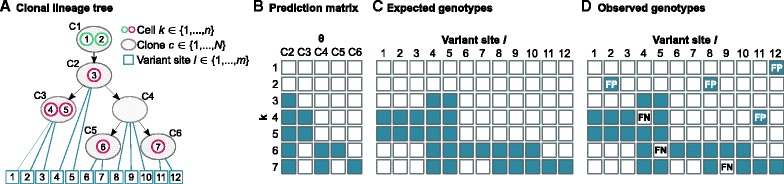
Toy example of the OncoNEM scoring model. **a** Hypothesis of a clonal lineage tree that describes the subpopulations of a tumor (*grey circles*) and their relationships (*black arrows*). **b** This tree can be represented as a prediction matrix that predicts the mutation pattern we expect to see across all *k* cells for a mutation that occurred in a certain clone *θ*. **c** Assuming that we know the originating clone of every mutation (*blue lines* in clonal lineage tree), we can extend the prediction matrix to a full matrix of expected genotypes. **d** To score the tree, expected genotypes are compared to observed genotypes. The more mismatches there are, the lower the likelihood of the tree given the data. Since the origin of a mutation is unknown a priori, the full likelihood of the lineage tree is calculated by marginalizing over all possible origins for every mutation. *FN* false negative, *FP* false positive

The OncoNEM scoring function is derived from nested effects models, which evaluate noisy subset relations in gene perturbation screens to infer signaling hierarchies [24, 25]. To model the accumulation of mutations, we assume that each locus gets mutated only once (infinite sites assumption [26]) and that mutations are never lost. Under these assumptions, direct relationships between clones imply that the mutations of the ancestral clone are a subset of the descendants’ mutations. To define the likelihood of a tree given the observed genotypes, OncoNEM predicts the expected mutation patterns based on the tree and then scores the fit between predicted and observed mutations patterns while probabilistically accounting for genotyping errors. A schematic illustration of the OncoNEM scoring model is shown in Fig. 1. The derivation of the scoring function is described in ‘Materials and methods’.

#### Searching the tree space for high-scoring models

**Fig. 2:**
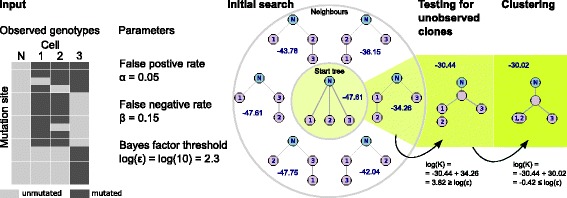
Toy example of OncoNEM inference steps. Given the observed genotypes and the input parameters *α* and *β*, the log-likelihood of the start tree, which is by default a star-shaped tree, is −47.61. In the first step of the initial search, all neighbors of the star tree are scored. The highest scoring tree obtained in this step has a log-likelihood of −34.26. In this toy example, the highest scoring tree of the first step is also the best cell lineage tree, overall. Therefore, the initial search terminates with this tree as a solution. In the first refinement step, we find that inserting an unobserved node into the branch point of our current tree increases the log-likelihood by 3.82. Since this improvement is larger than the Bayes factor threshold of 2.3, the solution with the unobserved clone is accepted. In the final refinement step, cells are clustered along edges. In the toy example, only one clustering step does not decrease the log-likelihood by more than log(*ε*)

OncoNEM inference is a three-step process. We start with an initial search, where we restrict the model space to cell lineage trees. This yields a first estimate of the tree and its likelihood. The second step tests whether adding unobserved clones to the tree substantially increases the likelihood. The third step yields the final model of the clonal lineage tree by clustering cells within the previously derived tree into clones. An overview of the inference steps is shown in Fig. 2 and details are described in ‘Materials and methods’.

### Simulation studies

We performed comprehensive simulations to assess the robustness of OncoNEM to errors in the parameter estimates, and compared its performance to six baseline methods. As representatives of classic phylogenetic methods we used likelihood optimization of neighbor joining trees, as applied by Hughes et al. [15], and Bayesian phylogenetic inference, as used by Eirew et al. [16]. Both methods yield solutions where each cell corresponds to a different leaf in the tree. This type of tree is not directly comparable to the simulated one. To at least be able to evaluate the clustering solutions of the two methods, we identified subpopulations of cells within these trees by hierarchical clustering of the trees’ distance matrices with silhouette-score-based model selection. As representatives of hierarchical clustering based methods and the approaches used by Gawad et al. [17] and Yuan et al. [18], we used hierarchical and k-centroids clustering with silhouette-score-based model selection and subsequent minimum spanning tree construction. Furthermore, we compared our method to BitPhylogeny [18] and a method for inferring oncogenetic trees by Kim and Simon [20].

For all but Kim and Simon’s method, clustering performance was assessed using the V-measure, whereas the overall tree reconstruction accuracy was measured using the pairwise cell shortest-path distance. Since Kim and Simon’s method neither infers the position of the sequenced cells within the tree nor performs any clustering, V-measure and single-cell shortest-path distance cannot be used to assess its performance. Instead we calculated the accuracy of the inferred mutation orders. See ‘Materials and methods’ for details of benchmarking measures and data simulation.

#### OncoNEM is robust to changes in error parameters *α* and *β*

**Fig. 3:**
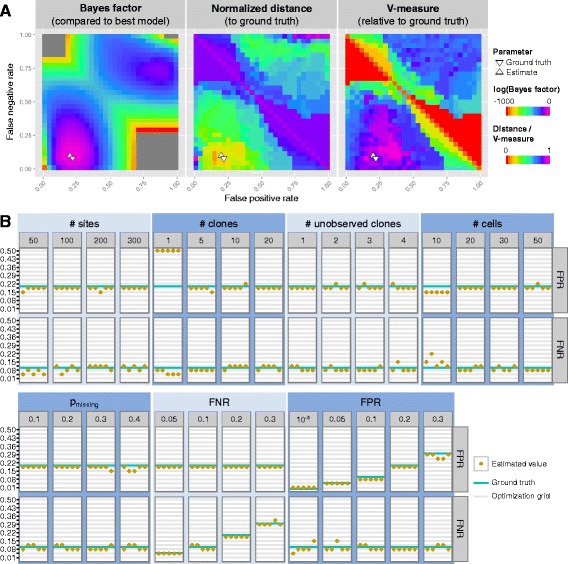
Parameter estimation. **a** Dependence of OncoNEM results on inference parameters. Log Bayes factor of highest scoring model inferred with given parameter combination relative to highest scoring model overall. The inferred parameters ($\hat {\alpha }=0.22$, $\hat {\beta }=0.08$) are close to the ground truth (*α*=0.2, *β*=0.1). A large range of parameter combinations around the ground truth parameters yield solutions close to the ground truth tree in terms of pairwise cell shortest-path distance and V-measure. The distance was normalized to the largest distance observed between any inferred tree and the ground truth. **b** Parameter estimation accuracy. FPRs and FNRs estimated by OncoNEM for various simulation settings with five replicates each. The *blue lines* mark the ground truth parameters. The *grey lines* mark the grid values over which FPR and FNR were optimized

To test if our method can infer the main model parameters, FPR *α* and FNR *β*, and to evaluate the robustness of our method to errors in those estimates, we simulated a tree containing ten clones, two of which were unobserved, with a total number of 20 cells. A corresponding genotype matrix with 200 SNVs was simulated using an FPR of 0.2, an FNR of 0.1 and 20 % missing values. Then, we inferred clonal lineage trees as described above, using various combinations of FNRs and FPRs, and compared the inferred trees to the ground truth. As Fig. 3a shows, a large range of parameter combinations yield solutions that are close to the original tree in terms of pairwise cell shortest-path distance and V-measure with both the inferred and the ground truth parameters lying in the middle of this range. Similar results were obtained on a second data set that was simulated using a much lower FPR of 10^−5^ (see Additional file 1: Figure S1). These results demonstrate that OncoNEM is robust to changes in the model parameters.

#### OncoNEM estimates model parameters accurately

In the second simulation study, we further assessed the parameter estimation accuracy of OncoNEM. To generate different test data sets, we varied simulation parameters such as noise levels, number of cells, number of mutation sites, number of clones, fraction of missing values and the number of unobserved clones.

With unknown error rates, we compared the estimated FPR and FNR to the ground truth parameters. As shown in Fig. 3b, the estimated parameters are close to the ground truth parameters for all but the single-clone case. This demonstrates that OncoNEM estimates model parameters accurately over a wide range of simulation settings.

#### OncoNEM is robust to changes in *ε*

**Fig. 4:**
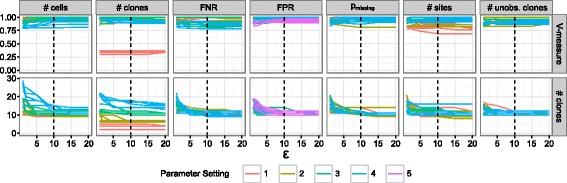
Dependence of OncoNEM’s clustering solution on Bayes factor threshold *ε*. This figure shows the V-measure and the number of clones of the OncoNEM solution as a function of *ε* for various simulation scenarios. Every *line* corresponds to one data set of the method comparison study. *Lines* are color coded by parameter setting for the varied simulation parameter. In all simulation scenarios, the number of clones is largely independent of *ε*, unless it is set to be unreasonably small (*ε*<5). The threshold *ε* used throughout the simulation and case studies is 10 (*dashed line*), and thus well within the stable range

Next, we assessed the sensitivity of OncoNEM to changes in the Bayes factor threshold *ε*. We applied OncoNEM to each simulated data set described in the previous section, using varying values for *ε* and recoded the inferred number of clones (see Fig. 4). In all simulation scenarios, the number of clones is largely independent of *ε*, unless this parameter is set to very low values (*ε*<5). Throughout all further simulation and case studies, *ε* was kept constant at 10, which is well within the stable range.

#### OncoNEM outperforms baseline methods

**Fig. 5:**
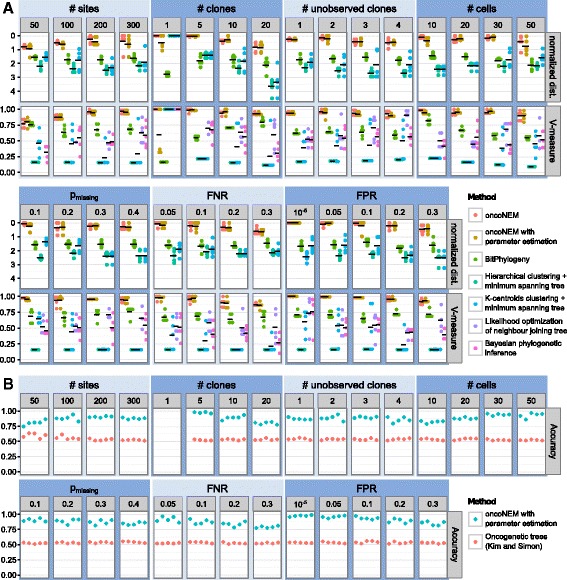
OncoNEM performance assessment. **a** Performance comparison of OncoNEM and five baseline methods. Shown are the distance and V-measure of inferred trees to ground truth. Results of single simulations are marked by *dots* and colored by method, while *black horizontal bars* indicate the mean over five simulations for each method. The distances shown were normalized for the number of cells *n* in the trees and were obtained by dividing the pairwise cell shortest-path distances by *n*(*n*−1)/2. Distances could only be calculated for three of the baseline methods. Values of the varied parameters are shown in the *panels at the top*. As default parameters, we used an FNR of 0.1, an FPR of 0.2, 200 sites, ten clones, no unobserved clones, 20 cells and 20 % missing values. **b** Performance comparison of OncoNEM and Kim and Simon’s oncogenetic tree method. Shown is the mutation order accuracy of the inferred trees for each of the simulated data sets. This measure is undefined for data sets without mutually exclusive mutations. Therefore, no values are shown for the single-clone case and the first replicate of the five-clone scenario, for which the simulated tree is linear

Finally, using the same simulated data as above, we compared the performance of OncoNEM with known and unknown inference parameters to the performance of the six baseline methods mentioned above. The results of the method comparison are shown in Fig. 5. OncoNEM substantially outperforms the other methods for all simulation scenarios but the single-clone case. It consistently yields results that have a smaller distance to the ground truth and a higher V-measure than the baseline methods or, for oncogenetic trees, infers the order of mutation with a much higher accuracy. Overall, OncoNEM’s performance with unknown model parameters is comparable to its performance with given parameters.

In summary, the simulation results demonstrate that OncoNEM clearly outperforms the baseline methods for the tested simulation scenarios even if the model parameters are unknown a priori.

### Case study 1: muscle-invasive bladder transitional cell carcinoma

We used OncoNEM to infer the evolutionary history of a muscle-invasive bladder transitional cell carcinoma previously analyzed by Li et al. [11], who performed single-cell exome sequencing of 44 tumor cells, as well as exome sequencing of normal and tumor tissue. Li et al. estimated the average ADO rate to be 0.4 and the FDR to be 6.7×10^−5^. Using a census-filtering threshold of 3, they identified 443 SSNVs across the 44 cells. In their final genotype matrix, 55.2 % of the values were missing.

**Fig. 6:**
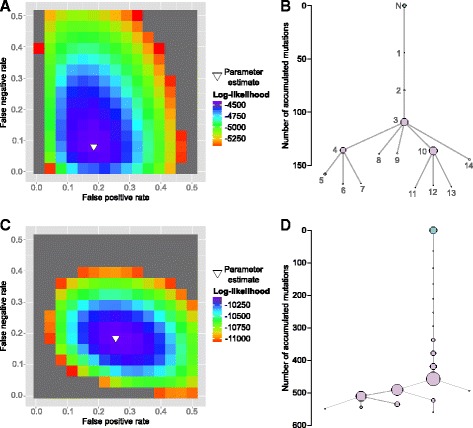
Case study results. **a**, **b** Results inferred by OncoNEM on bladder cancer data set. The estimated error rates are *α*=0.185 and *β*=0.08. The inferred tree suggests a branching evolution with three major subpopulations. **c**, **d** Results inferred by OncoNEM on the essential thrombocythemia data set. The estimated error rates are *α*=0.255 and *β*=0.185. The inferred tree suggests a largely linear evolution with some small subpopulations branching off late during tumor evolution

We binarized the genotype matrix by setting homozygous normal sites to 0 and hetero- or homozygous mutant sites to 1 and applied OncoNEM as described above. The resulting tree is shown in Fig. 6b. The single linear branch from the normal suggests that all cells in the data set are descendants of a single founder cell. The tree contains three major subpopulations. The least mutated of these subpopulations carries about a quarter of the detected mutations. These trunk mutations are shared by almost all of the analyzed cells. This early clone gave rise to multiple divergent subpopulations, two of which are large and again diversified into smaller subclones.

These results agree with the results of Li et al. who inferred three main subpopulations (A, B, C) with B and C having evolved from A. However, mapping the clone labels of Li et al. onto the OncoNEM tree shows that the assignment of cells to clones differs between the two approaches (see Additional file 1: Figure S2). Li et al. also inferred the origins of eight mutations in seven genes that are commonly altered in muscle-invasive bladder transitional cell carcinomas. A comparison of their results with the posterior probability of *θ* inferred by OncoNEM is shown in Table 1. The assignment of mutations to clones agrees in seven out of eight cases.

**Table 1:** Comparison of origin of mutations inferred by OncoNEM with origins inferred by Li et al.

		1	2	3	4	5, 7	6	10	11, 12	13	8, 9, 14
A	*NIPBL*	***0.33***	***0.33***	***0.33***	0.00	0.00	0.00	0.00	0.00	0.00	0.00
	*CFTR*	**0.45**	**0.45**	**0.09**	0.00	0.00	0.00	0.00	0.00	0.00	0.00
	*DHX57*	**0.45**	**0.45**	**0.09**	0.00	0.00	0.00	0.00	0.00	0.00	0.00
	*ASTN1*	**0.25**	**0.25**	**0.51**	0.00	0.00	0.00	0.00	0.00	0.00	0.00
B	*ATM*	0.00	0.00	0.00	**1.00**	**0.00**	**0.00**	0.00	0.00	0.00	0.00
C	*COL6A3*	0.07	0.07	0.07	0.00	0.00	0.00	**0.76**	**0.01**	**0.00**	0.00
	*KIAA1958* ^1^	0.00	0.00	0.00	0.00	0.00	0.00	**0.98**	**0.00**	**0.00**	0.00
	*KIAA1958* ^2^	0.19	0.19	0.19	0.02	0.00	0.02	**0.38**	**0.00**	**0.00**	0.00

Posterior probabilities of *θ* inferred by OncoNEM for the eight recurrently mutated genes analyzed by Li et al. A, B and C denote the clones inferred by Li et al., and 1 to 14 denote the clones inferred by OncoNEM. Visual comparison of the OncoNEM tree with the phylogeny inferred by Li et al. suggests that clone A corresponds to clones 1–3, clone B corresponds to clones 4–7 and clone C corresponds to clones 10–13 (indicated in bold). Overall, both methods assign mutations to the same clones. *KIAA1958* ^1^ denotes mutation at chromosome 9, position 114376732. *KIAA1958* ^2^ denotes mutation at chromosome 9, position 114376902

OncoNEM estimated the FPR to be 0.185 (see Fig. 6a). This error rate is higher than the expected value under the binomial model used for consensus filtering by Li et al., which suggests that there might be recurrent sequencing errors in the data set. The FNR was estimated to be 0.08. This estimated value lies within the expected range of less than half the estimated ADO rate. See the parameter estimation section within ‘Materials and methods’ for an explanation of the conceptual differences between the original error rates estimated by Li et al. and the OncoNEM parameters.

To test the robustness of our results, we inferred trees using model parameters that are slightly different from the estimated ones (see Additional file 1: Figure S3). The structure and the overall features of the resulting trees are close to the original estimate, which further supports our results.

#### Impact of loss of heterozygosity on inference results

The OncoNEM model assumes that mutations are never lost. Deletions that lead to loss of heterozygosity (LOH) are, however, common in various types of cancer.

We expect that our algorithm is able to infer good solutions despite LOH events, as long as the fraction of mutations affected by LOH is relatively small. In this case, LOH-affected sites will simply contribute to the error rates of false positives and false negatives, depending on whether the deletion occurred early or late after the original occurrence of the SNV.

To support this claim, we identified the LOH-affected regions of the bladder cancer from a bulk-sequencing analysis by Li et al. (see Additional file 1: Table S1) and removed all mutations within these regions from the mutation data set (6.3 % of all variant sites). We then applied OncoNEM to this reduced data set and compared the solution to the one obtained from the full data set. Additional file 1: Figure S4 shows that the inferred tree is largely stable and the overall tree structure remains the same.

### Case study 2: essential thrombocythemia

In the second case study, we applied OncoNEM to a data set derived by single-cell exome sequencing of 58 single cells from an essential thrombocythemia [10]. Hou et al. estimated the average ADO rate to be 0.42 and the FDR to be 6.4×10^−5^. Using a census-filtering threshold of 5, they identified 712 SSNVs. Their final genotype matrix contained 57.7 % missing values.

The genotypes were binarized and OncoNEM was applied as in the previous case study. The inferred tree is shown in Fig. 6d. Again, the tree suggests that all tumor cells are descendants of a single founder cell. The majority of cells belong to subpopulations that are related through a linear trajectory. All detected branching events have occurred late during tumor development, i.e., after the tumor had already acquired more than 60 % of its mutations.

These results agree with the somatic mutant allele frequency spectrum analysis of Hou et al. that suggests that the neoplasm is of monoclonal origin [10], while Kim and Simon inferred a mutation tree with a complex hierarchy [20]. Using BitPhylogeny, Yuan et al. [18] inferred a polyclonal origin. However, with 58 cells, the data set might be too small for their method to converge.

OncoNEM estimated the FPR and FNR to be 0.255 and 0.185, respectively. The FPR estimate is again higher than expected under the binomial model, whereas the FNR lies within the expected range. As in the previous case study, running OncoNEM with similar parameters yields similar trees (see Additional file 1: Figure S5).

Given the error rates inferred by OncoNEM, the log-likelihood of the BitPhylogeny tree computed under the OncoNEM model is −11584, whereas the OncoNEM tree has a log-likelihood of −9964. The fact that the OncoNEM solution has a much higher likelihood than the BitPhylogeny tree shows that the differences are not due to the heuristic nature of OncoNEM’s search algorithm, but instead suggest that BitPhylogeny did not converge to the optimal solution.

These two case studies showed how OncoNEM can extend and improve on previous analyses of these data sets.

## Conclusions

OncoNEM is an accurate probabilistic method for inferring intra-tumor phylogenies from noisy observations of SSNVs of single cells. It is based on the nested structure of mutation patterns of phylogenetically related cells. The input to our method is a binary genotype matrix, which may contain missing values as well as false positives and false negatives. OncoNEM identifies subpopulations within a sample of single cells and estimates their evolutionary relationships and underlying genotypes, while accounting for the high error rates of single-cell sequencing. OncoNEM can estimate model parameters directly from the input data and is robust to changes in those estimates.

In simulations, OncoNEM performs well for error rates of current single-cell data sets and large fractions of missing values, and substantially outperforms baseline methods. We have applied OncoNEM in two case studies, showing that the OncoNEM results agree with previous results, which were based on manual inference and the analysis of somatic mutant allele frequency spectra, while also providing a more refined picture of the tumors’ histories. In one case study, we have also shown that OncoNEM yields robust results even if parts of the genome are affected by LOH.

Our general recommendation is to blacklist LOH-affected regions before OncoNEM inference, if additional data like bulk-sequencing is available. If the evolution of the tumor is known to be copy number driven and LOH affects very large parts of the genome, we recommend using a copy-number-based method for inferring tumor evolution.

OncoNEM can easily be applied to single-cell data sets of current size. For much larger data sets, the current search algorithm may become too computationally expensive. Currently the model cannot be used for copy number variations, which are not independent of each other and show horizontal dependencies [27] and we plan to extend the model to this data type in the future.

Recent advances have made it possible to sequence both the genome and transcriptome of a single cell [28, 29]. In the future, this will allow us to combine single-cell phylogenies with single-cell transcriptomics to gain insights into how the expression of genes changes as a tumor evolves.

In summary, OncoNEM is a major step towards understanding the clonal evolution of cancer at single-cell resolution.

## Materials and methods

### Likelihood of a clonal lineage tree

#### Data

We assume that the variants of the single cells have already been called and filtered so that the data set only contains the somatic variant sites. Let *D*=(*d*_*kl*_) be the matrix of observed genotypes where *k*∈{1,…,*n*} is the label of a single cell and *l*∈{1,…,*m*} is the index of a mutation site. Let *d*_*kl*_∈{0,1,NA} denote the mutation status of cell *k* at site *l*, where 0, 1 and NA encode an unmutated, mutated or unknown site, respectively.

#### Clonal lineage trees

We assume that a clonal lineage tree is a directed not necessarily binary tree $\mathcal {T}$ whose root is the unmutated normal. Every node of this tree represents a clone *c*∈{1,…,*N*} that contains 0, 1 or multiple cells of the data set. Let *c*(*k*) denote the clone that contains cell *k*. In the following, we assume without loss of generality that the root has index 1.

#### OncoNEM

An OncoNEM has two parts: the clonal lineage tree $\mathcal {T}$ and the occurrence parameter $\Theta =\left \{\theta _{l}\right \}_{l=1}^{m}$, where *θ*_*l*_ takes the value *c* of the clone where mutation *l* originated.

The core of our method is a function that defines the probability of the OncoNEM given a data set *D* and is derived in the following. Using a Bayesian approach, the posterior probability of $\mathcal {T}$ and *Θ* given *D* can be written as

(1) $$ P(\mathcal{T},\Theta|D) = \frac{P(D|\mathcal{T},\Theta) \, P(\Theta|\mathcal{T}) \, P(\mathcal{T})}{P(D)}.  $$

The model prior $P(\mathcal {T})$ can be used to incorporate prior biological knowledge. We assume it to be uniform over the search space. The normalizing factor *P*(*D*) is the same for all models and it is not necessary to compute it when comparing them. Therefore,

(2) $$ P(\mathcal{T},\Theta|D) \propto P(D|\mathcal{T},\Theta) \, P(\Theta|\mathcal{T}).  $$

#### Likelihood for known *Θ*

Let us assume that we know for each locus *l* in which clone the mutation occurred and that no mutations occur in the normal. This is equivalent to restricting the parameter space of *θ*_*l*_ to {2,…,*N*} and is justified by stringent variant filtering of the input data.

Given $\mathcal {T}$ and *Θ*, we can predict the genotype of every cell: if *c* is the clone in which a mutation occurred, the mutation is present in *c* and all descendants of *c* and absent in all other clones, i.e., given *θ*_*l*_=*c*, the tree determines the predicted genotype *δ*_*kl*_.

Finally, to calculate the likelihood of $(\mathcal {T},\Theta)$, we compare the expected genotypes with the observed ones. We model the genotyping procedure as draws of binary random variables *ω*_*kl*_ from the sample space *Ω*={0,1} and assume that, given $\mathcal {T}$ and *Θ*, the random variables are independent and identically distributed according to the probability distribution

(3) $$ P\left(\omega_{kl} | \delta_{kl}\right) =\left(\begin{array}{ll} P\left(0 | 0\right) & P\left(1 | 0\right)\\ P\left(0 | 1\right) & P\left(1 | 1\right) \end{array} \right) = \left(\begin{array}{cc} 1-\alpha & \alpha \\ \beta & 1-\beta \end{array} \right),  $$

where *α* and *β* are global probabilities of false positive and false negative draws, respectively.

We interpret the observed genotypes *d*_*kl*_ as events from the event space $\mathcal {P}(\Omega) = \{\emptyset,\{0\},\{1\},\{0,1\}\}$, where a missing value corresponds to the event {0,1}. Then, the probability of the observed genotypes *D* given $\mathcal {T}$ and *Θ* is

(4) $$ P(D|\mathcal{T},\Theta) = \prod\limits_{l=1}^{m} \prod\limits_{k=1}^{n} P(\omega_{kl} \in d_{kl} | \delta_{kl}),  $$

where

(5) $$ P\left(\omega_{kl} \in d_{kl} | \delta_{kl}\right) =\left\{ \begin{array}{ll} 1-\alpha & \text{if}~ d_{kl}=\{0\}~ \text{and}~ \delta_{kl}=0 \\ \alpha & \text{if}~ d_{kl}=\{1\}~ \text{and}~\delta_{kl}=0 \\ \beta & \text{if}~ d_{kl}=\{0\}~ \text{and}~ \delta_{kl}=1 \\ 1-\beta & \text{if}~ d_{kl}=\{1\}~\text{and}~ \delta_{kl}=1 \\ 1 & \text{if}~ d_{kl}=\{0,1\} \end{array} \right.  $$

is the probability of a single observation given the predicted genotype.

#### Likelihood for unknown *Θ*

So far we assumed *Θ* to be known, but this is generally not the case. To derive the likelihood of the entire data matrix, we treat *Θ* as a nuisance parameter and marginalize over it. Furthermore, we make two assumptions: First, the occurrence of one mutation is independent of the occurrence of all other mutations, i.e.,

(6) $$  P(\Theta|\mathcal{T}) = \prod\limits_{l=1}^{m} P(\theta_{l}|\mathcal{T}),  $$

and second, the prior probability of a mutation occurring in a clone is

(7) $$ P(\theta_{l}=c|\mathcal{T}) =\left\{ \begin{array}{ll} 0 & \text{if}~\textit{c}~\text{is the normal}~ (c=1), \\ \frac{1}{N-1} & \text{otherwise}. \end{array} \right.  $$

Then the marginal likelihood is

(8) $$ \begin{aligned} P(D|\mathcal{T}) =& \int P(D|\mathcal{T},\Theta) P(\Theta|\mathcal{T}) \mathrm{d}\Theta \\ =& \frac{1}{(N-1)^{m}} \prod\limits_{l=1}^{m} \sum\limits_{c=2}^{N} \prod\limits_{k=1}^{n} P\left(\omega_{kl} \in d_{kl} |\mathcal{T},\theta_{l}=c\right) \\ =& \frac{1}{(N-1)^{m}} \prod\limits_{l=1}^{m} \sum\limits_{c=2}^{N} \prod\limits_{k=1}^{n} P\left(\omega_{kl} \in d_{kl} | \delta_{kl}\right). \end{aligned}  $$

### Algorithms to infer OncoNEMs

OncoNEM inference is a three-step process of initial search, testing for unobserved clones and clustering.

#### Step 1. Initial search: building a cell tree

The search space of cell lineage trees with *n* nodes contains *n*^*n*−2^ models, making exhaustive enumeration infeasible for trees with more than nine nodes. Therefore, we implemented a heuristic local search (see Algorithm 1), which avoids getting trapped in local optima by returning to neighbors of high-scoring previous solutions.



#### Step 2. Refinement: testing for unobserved clones

The number of sequenced single cells is usually small compared to the tumor size. Consequently, some clones of the tumor may not be represented in the single-cell sample. This problem is similar to the ‘unknown unknowns’ problem in reconstructing biological pathways [30], where latent variables that cause additional patterns in the observed data set can be inferred. In the OncoNEM setting, unobserved clones with at least two child clones create additional mutation patterns and can, therefore, potentially be inferred. OncoNEM accounts for this possibility by testing if there is a lineage tree with additional, unobserved branch nodes that can better explain the observed data (see Algorithm 2). Unobserved clones that linearly connect observed clones cannot be inferred, but they also do not change the shape of the tree.



Briefly, the algorithm generates trees with *n*+1 nodes from the previous solution by inserting an unobserved node into its branch points. These trees are used as start trees in a new search that optimizes the position of the unobserved node in the tree. A larger model is accepted if the Bayes factor of the larger versus the smaller model is larger than a threshold *ε* (see below). If the larger model passes the threshold, these expansion steps are repeated, otherwise the algorithm terminates with the smaller solution.

#### Step 3. Refinement: clustering cells into clones

The clustering procedure tests if the data can be explained better or equally well by a clonal lineage tree in which multiple cells correspond to the same node (see Algorithm 3). Nodes are clustered iteratively along branches until merging cells into clones decreases the likelihood by more than a factor of 1/*ε* compared to the best clustering solution found so far. Cells may be clustered into clones because they are genetically very similar or because of the limited information content of the data, which can be due to genotyping errors, missing values or a restricted number of SSNVs in the sequenced regions of the genome.



#### Choosing the Bayes factor threshold ***ε***

Choosing the parameter *ε* is a trade-off between declaring clones with little support from the data and overly strict clustering. In this setting, choosing *ε*>1 means that we prefer the smaller model unless the strength of evidence for the larger model compared to the smaller one exceeds a certain threshold. Jeffreys’s [31] or Kass and Raftery’s [32] scale for the interpretation of the Bayes factor can be used as guidance. We used a value of *ε*=10, which denotes strong evidence according to Jeffreys’s scale.

### Estimating *Θ*, the occurrence of mutations

Given a lineage tree, we can estimate which clones acquired which mutations during tumor development. To do this, we calculate the posterior probability of a mutation having occurred in clone *c*. Using a uniform prior for the occurrence parameter *θ*_*l*_∈{2,…,*N*}, we obtain

(9) $$ P(\theta_{l}=c | \mathcal{T},D)=\frac{1}{Z} \prod\limits_{k=1}^{n} P\left(\omega_{kl} \in d_{kl} | \mathcal{T},\theta_{l}=c\right),  $$

with normalizing constant

(10) $$ Z = \sum\limits_{c=2}^{N} \prod\limits_{k=1}^{n} P\left(\omega_{kl} \in d_{kl}|\mathcal{T},\theta_{l}=c\right).  $$

The branch lengths *L* of the tree can be estimated as the expected number of mutations that separate a clone *c* from its parent pa(*c*),

(11) $$ L_{\text{pa}(c),c} = \sum\limits_{l=1}^{m} P(\theta_{l}=c|\mathcal{T},D).  $$

### Estimating model parameters *α* and *β*

Previous studies have estimated FDRs and ADO rates from the sequencing data [9, 10]. These error rates are, however, not equivalent to the error parameters FPR *α* and FNR *β* used by OncoNEM. This is due to three pre-processing steps that are applied to the sequencing data to generate the final genotype matrix.

In the first step, only sites that appear to be mutated are selected. Selecting only sites that report mutations from all sequenced sites enriches for false positives. It also means that the FPR used by OncoNEM is conceptually very different from the FDR reported in these studies. The FPR describes what fraction of truly non-mutant sites is reported as mutant in the observed genotype matrix, whereas the FDR corresponds to the number of false positive variants per sequenced base pair.

Even with a very small FDR, the total number of false positive variants is expected to be large, because the sequenced exome is very large. Therefore, the second pre-processing step is consensus-based variant filtering, which only selects mutations that occur multiple times for the final data set. Li et al. [11] selected the census-filtering threshold so that, under a binomial model, no site is expected to be non-mutant in all cells. However, this step cannot remove recurrent false positives caused by systematic sequencing errors. In addition to changing the FPR, this step also reduces the FNR, as it preferentially removes sites that have an above-average ADO rate.

Thirdly, a binarization step is performed that interprets all homozygous mutant sites as heterozygous normal/mutant. This step reduces the FNR by approximately 50 % and further explains why the FDR is expected to differ from previously estimated ADO rates.

While all of these steps are expected to change the error rates of the final data set, the exact impact on the parameters is difficult to estimate. Therefore, we chose to estimate error rates for our model directly from the data.

We treat the selection of model parameters as part of the learning problem and estimate them using a maximum likelihood approach, similar to Zeller et al. [33]. We create a grid of parameter combinations *α* and *β* and optimize $\mathcal {T}$ given these parameters using the heuristic search algorithm. Then, we choose the parameter combination that yields the highest scoring tree and infer a clonal lineage tree as described above.

This parameter estimation process is computationally expensive compared to the tree inference. However, it can easily be parallelized and the grid of parameter combinations can be coarse as OncoNEM is robust to changes in the model parameters around the optimum (see simulation results). Furthermore, the range of tested parameter combinations can be reduced in the presence of prior knowledge.

### Data simulation

For the simulation study, data sets were created in a two-step procedure that consists of (1) generating a tree structure and (2) simulating the corresponding genotypes.

#### Simulating clonal lineage trees

To simulate a tree with *c* clones, we select clone one to be the root and the parent of the second clone. Then, the remaining clones are added iteratively by choosing a non-root node that is already part of the tree with uniform probability as parent.

When simulating trees with unobserved clones, we count how many nodes in the simulated tree have at least two children. If this number is greater than or equal to the desired number of unobserved clones *c*_*u*_, we randomly choose *c*_*u*_ of these nodes as unobserved clones, otherwise a new tree is simulated. Next, we assign one cell to every observed clone. For the remaining cells, clones are chosen iteratively with a probability proportional to the current clone size, to generate clones of different sizes.

#### Simulating genotype observations

For every mutation site, we choose the occurrence parameter *θ*_*l*_ with uniform probability from all non-root nodes. Given *Θ* and the tree structure, the full matrix of true genotypes is obtained by setting an entry to 1, if the mutation occurred in a clone that is ancestral to the cell’s clone or if the mutation occurred in the clone containing the cell itself, and 0 otherwise.

Observed genotypes are derived from true genotypes by (1) setting a fraction *p*_missing_ of randomly chosen values to NA, (2) setting a fraction *α* of unmutated, non-missing entries to 1 and (3) setting a fraction *β* of mutated, non-missing entries to 0. If this yields sites without any observed mutations, we add, for each of these sites, a false positive to a randomly chosen cell. Finally, to avoid a bias in the method testing, we randomize the order of cells in the matrix of observed genotypes.

### Comparison measures for method benchmarking

Clustering performance was assessed using the V-measure [34], an entropy-based cluster evaluation measure that assesses both completeness and homogeneity of the clustering solution. The V-measure takes values from 0 to 1, with higher values indicating a better performance.

**Fig. 7:**
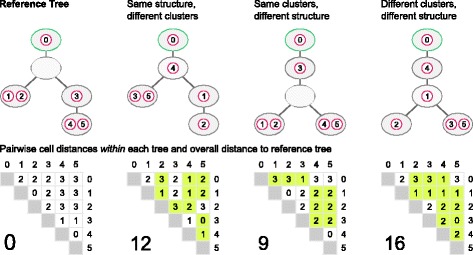
Comparing clonal trees with the pairwise cell shortest-path distance. The *yellow* entries in the pairwise distance matrices indicate differences from the reference tree

To assess the similarity between trees, we developed a distance measure called *pairwise cell shortest-path distance* (see Fig. 7). Given are two trees, $\mathcal {T}_{1}$ and $\mathcal {T}_{2}$, built on the same set of cells {1,…,*n*}, but potentially differing in the number of nodes (clones). Note that the root of a tree can be an empty node. To ensure that every node of the tree is taken into account in the distance measure, we add an extra cell to the root before calculating the distance. Without loss of generality, we denote this additional cell in the root node with index 0. For every pair of cells *i* and *j*, we compute the shortest-path *d*_*ij*_(·) between the two cells in each tree. If the two cells belong to the same clone, their shortest-path distance is 0, otherwise the shortest-path distance equals the number of edges (regardless of direction) that separate the clones of the two cells. Finally, we sum up the absolute differences between the shortest-path distances of all unordered pairs of cells in the two trees to obtain the overall pairwise cell shortest-path distance:

(12) $$ d(\mathcal{T}_{1},\mathcal{T}_{2}) = \sum\limits_{i=0}^{n-1} \sum\limits_{j=i+1}^{n} | d_{ij}(\mathcal{T}_{1}) - d_{ij}(\mathcal{T}_{2})|.  $$

A proof that this distance is a metric can be found in Additional file 1.

We define the *mutation order accuracy* of a tree $\mathcal {T}_{1}$ given the ground truth tree $\mathcal {T}_{2}$ as the average of

- the fraction of correctly inferred pairwise mutation orders, i.e., the probability that mutation a is upstream of mutation b in $\mathcal {T}_{1}$ given that a is upstream of b in $\mathcal {T}_{2}$, and
- the fraction of correctly inferred mutually exclusive mutations, i.e., the probability that two mutations a and b lie on separate branches in $\mathcal {T}_{1}$ given that a and b lie on separate branches in $\mathcal {T}_{2}$

for all mutations that belong to different clusters in $\mathcal {T}_{2}$.

### Software and data availability

OncoNEM has been implemented in R [35] and is freely available under a GPL3 license on bitbucket [36]. Additional file 2 is a Knitr file reproducing all figures of the simulation studies. Additional file 3 is a Knitr file reproducing all figures of the case studies. Additional files 4 and 5 are the corresponding PDF files.

The processed single-cell data sets are provided in the OncoNEM R package. The sequencing data from both single-cell studies are deposited in the NCBI Sequence Read Archive [37]. The accession numbers are [SRA:SRA051489] for the bladder cancer study [11] and [SRA:SRA050202] for the essential thrombocythemia study [10].

## Ethics approval

Ethics approval was not needed for this study.

## Additional files

Additional file 1 Supplementary information. A PDF file containing five supplementary figures, one supplementary table, the definition of the pairwise cell shortest-path distance and a proof showing that this distance is a metric. (PDF 631 kb)

Additional file 2 Knitr file for simulation studies. An rnw file for reproducing the results of the simulation studies. (RNW 58.3 kb)

Additional file 3 Knitr file for case studies. An rnw file for reproducing the results of the case studies. (RNW 27.7 kb)

Additional file 4 PDF of compiled knitr script for simulation studies. A PDF file reproducing the results of the simulation studies. (PDF 594 kb)

Additional file 5 PDF of compiled knitr script for case studies. A PDF file reproducing the results of the case studies. (PDF 463 kb)

## Abbreviations

ADO: allele dropout
FNR: false negative rate
FPR: false positive rate
LOH: loss of heterozygosity
SNV: single nucleotide variant
SSNV: somatic single nucleotide variant
