# Supplementary material for: OncoNEM: inferring tumor evolution from single-cell sequencing data
Source: Genome Biol. 2016 Apr 15;17:69. doi: 10.1186/s13059-016-0929-9 (PMC4832472; doi:10.1186/s13059-016-0929-9)
Supplement: Additional file 1 — Supplementary information. A PDF file containing five supplementary figures, one supplementary table, the definition of the pairwise cell shortest-path distance and a proof showing that this distance is a metric. (PDF 631 kb) [file 13059_2016_929_MOESM1_ESM.pdf]

Supplementary information for OncoNEM: Inferring tumour evolution from single-cell sequencing data

Edith M Ross and Florian Markowetz

March 11, 2016

Supplementary figures

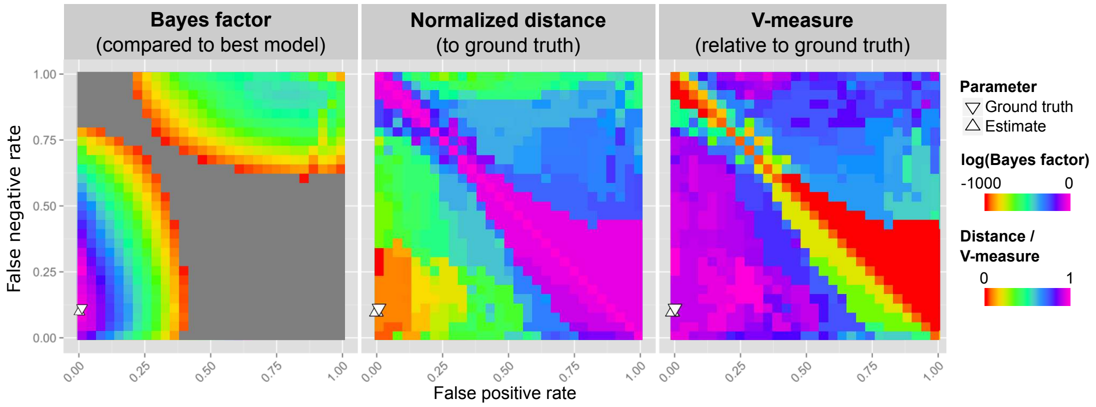

Figure S1: **Dependence of oncoNEM on inference parameters** This second example uses a much lower FPR of 10^{-5} and shows again that (i) oncoNEM estimates error parameters that are close to the ground truth parameters and (ii) oncoNEM is robust to changes in those parameters. The left panel shows the log Bayes factor of the highest scoring model inferred with the respective parameter combination relative to highest scoring model overall. The second and the third panels show that a large range of parameter combinations around the ground truth parameters yield solutions close to the ground truth tree in terms of pairwise cell shortest-path distance and V-measure. The distance was normalized to the largest distance observed between any inferred tree and the ground truth.

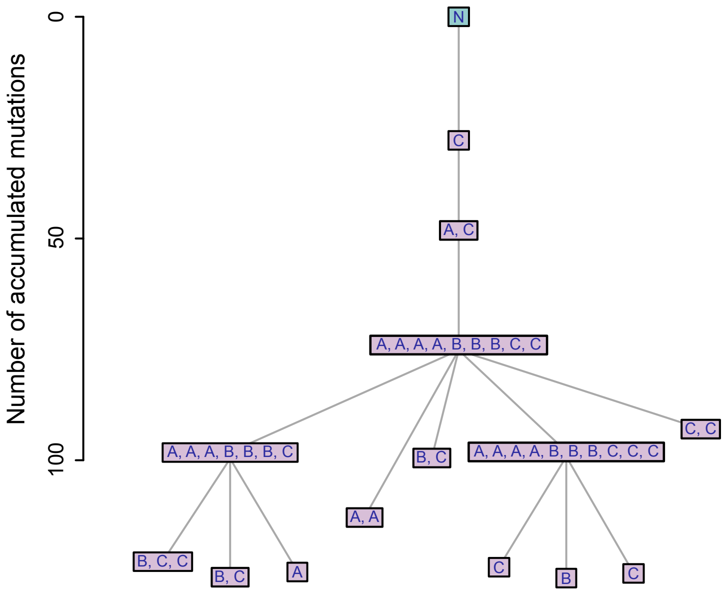

Figure S2: **Mapping of clone labels assigned by Li *et al* [1] onto cells in oncoNEM tree.** Comparison of clone labels between the two trees shows that the assignment of cells to clones differes between oncoNEM and the results by Li *et al*.

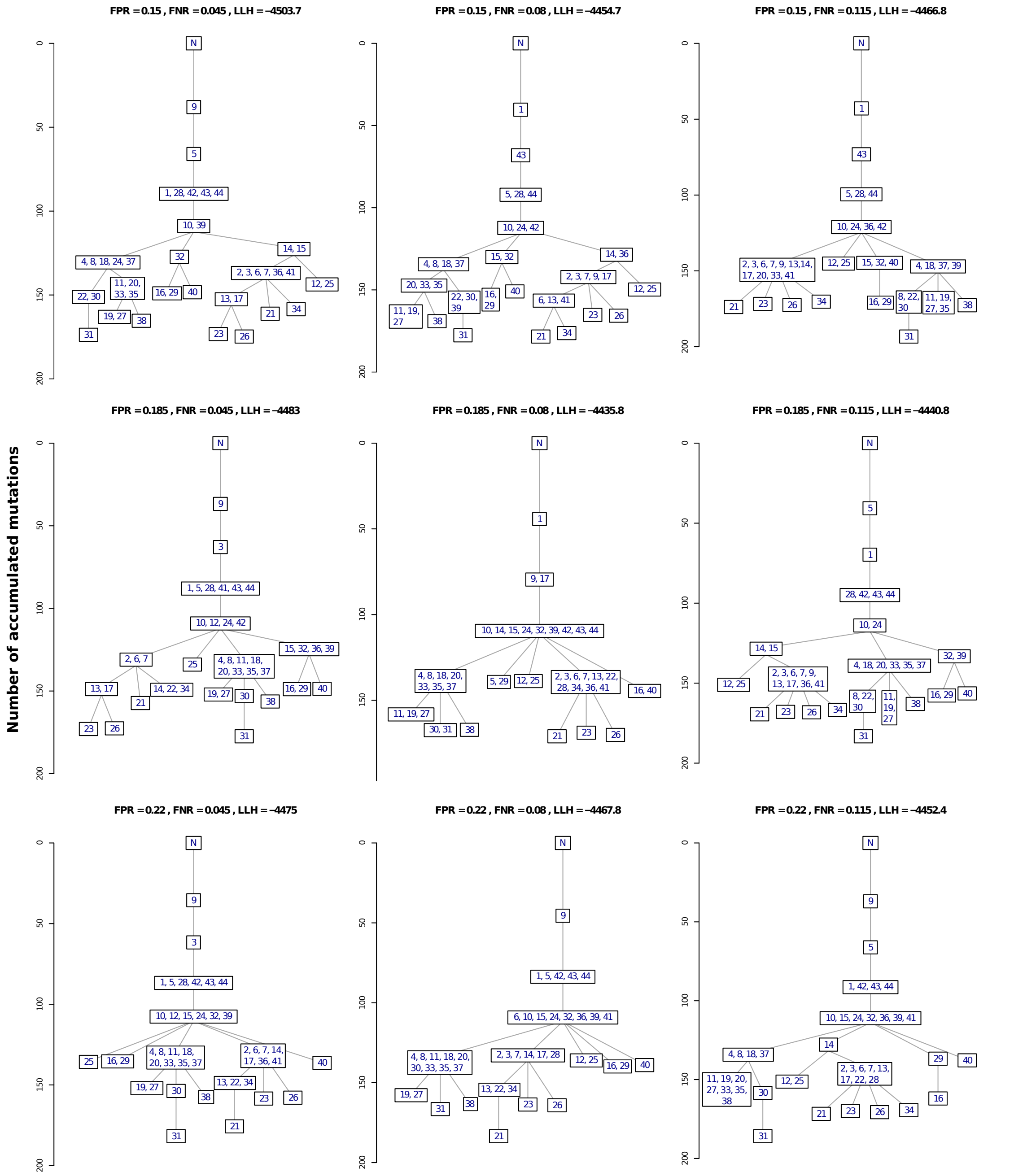

Figure S3: **Trees inferred with estimated parameters (middle tree) and parameters that are similar to the estimated ones (outer trees) for data set by Li *et al* [1].** Even if the inference parameters are varied, the overall stucture and features of the oncoNEM tree are preserved.

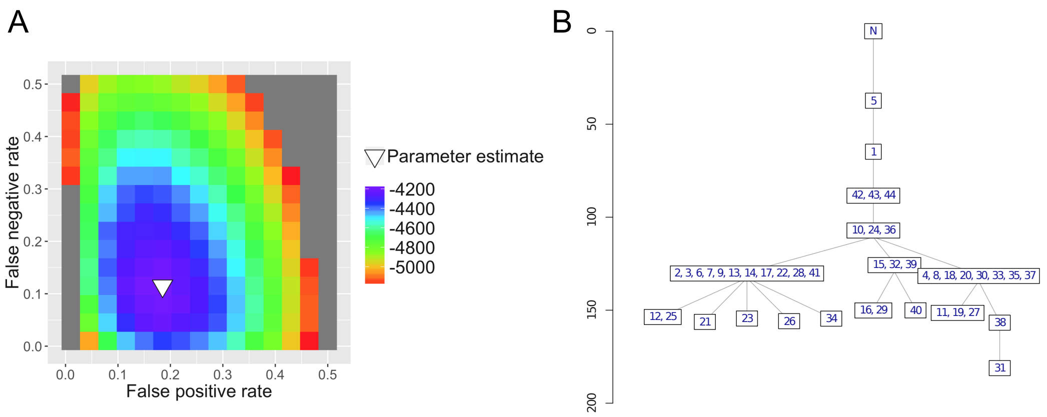

Figure S4: **OncoNEM solution based on a subset of the mutations in the bladder cancer data set.** For this analysis all mutations within regions affected by loss of heterozygosity were excluded from the data set. Genomic regions of the bladder cancer affected by loss of heterozygosity are shown in Table S1. Panel A shows the likelihood landscape with the inferred error parameters (FPR = 0.185, FNR = 0.115), which is close to the parameters estimated for the full data set. Panel B shows the inferred tree. As for the complete data set, the result suggests that initially the tumour underwent a linear evolution and then branched into two major sopopulations and some smaller ones.

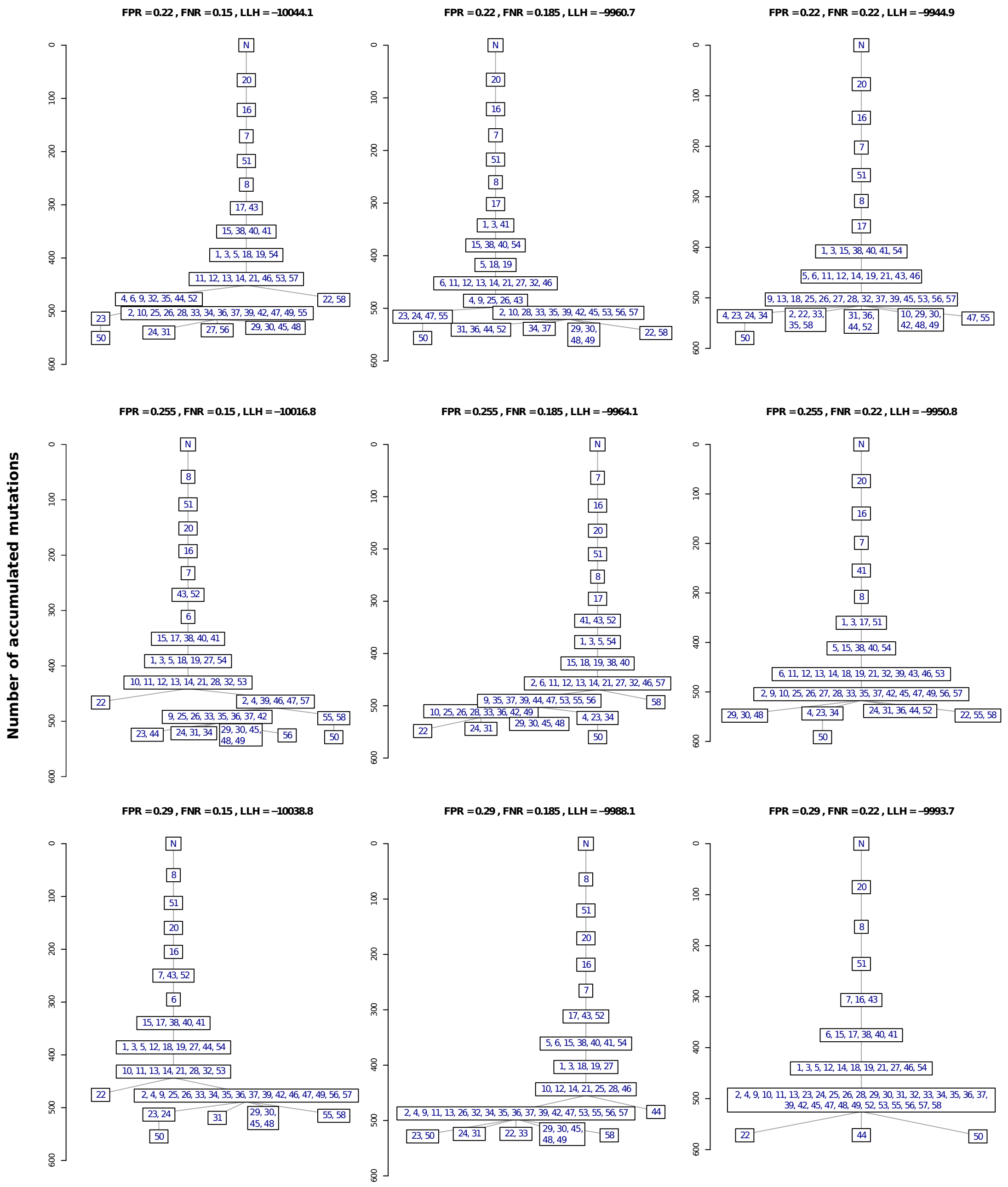

Figure S5: **Trees inferred with estimated parameters (middle tree) and parameters that are similar to the estimated ones (outer trees) for data set by Hou *et al* [2].** Even if the inference parameters are varied, the overall stucture and features of the oncoNEM tree are preserved.

Table S1: **Genomic regions of** This table summarizes all large genomic regions of the bladder cancer that are affected by loss of heterozygosity as shown in Figure S5 of Li *et al* [1]. To assess the effect of loss of heterozygosity on the oncoNEM result, the onoNEM inference was repeated on a subset of the original mutation data containing only SNVs that lie outside these regions.

**Chr**	**Region**	**Position (bp)**
2	q33.3 - q37.3	>205 600 000
9	entire chromosome	
10	q25.3 - q26.3	>114 900 000
11	p	<52 900 000
22	q	>11 800 000

Pairwise cell shortest-path distance

Definition Let $\mathcal{T}_1$ and $\mathcal{T}_2$ be two trees on the same set of cells $1, \ldots, n$ in which all leaf nodes contain at least one cell. In order to mark the root and to ensure that every node of the tree is taken into account in the distance measure, we add an extra cell with index 0 to the root. For two cells i and j in $0, \ldots, n$ let $d_{ij}(\mathcal{T}_.)$ be the number of edges separating the clones $c(i)$ and $c(j)$ in tree $\mathcal{T}_.$. With this we define the *pairwise cell shortest-path distance* between two trees $\mathcal{T}_1$ and $\mathcal{T}_2$ as

$$d(\mathcal{T}_1, \mathcal{T}_2) = \sum_{i=0}^{n-1} \sum_{j=i+1}^{n} |d_{ij}(\mathcal{T}_1) - d_{ij}(\mathcal{T}_2)|.$$

In order to show that this distance is a metric, we first prove the following lemma.

Lemma If $d_{ij}(\mathcal{T}_1) = d_{ij}(\mathcal{T}_2)$ for $\forall\, i, j \in \{0, \ldots, n\}$, then $\mathcal{T}_1 = \mathcal{T}_2$.

Proof We prove this by showing that, if $d_{ij}(\mathcal{T}_1) = d_{ij}(\mathcal{T}_2)$ for all i, j in $0, \ldots, n$, we can define a bijection $\phi : V(\mathcal{T}_1) \mapsto V(\mathcal{T}_2)$ that maps the vertices of $\mathcal{T}_1$ to $\mathcal{T}_2$ so that

(I) cell i is in clone v_j in tree $\mathcal{T}_1$ $\Leftrightarrow$ cell i is in clone $\phi(v_j)$ in tree $\mathcal{T}_2$ and

(II) $v_i = \text{pa}(v_j) \Leftrightarrow \phi\,(v_i) = \phi\,(\text{pa}(v_j))$, where $\text{pa}(v_j)$ is the parent of node v_j.

We construct the bijection in several steps.

(I) Assignment of observed clones.
The distance matrix uniquely defines the observed clones of a tree: Two cells i and j belong to the same clone if and only if $d_{ij}(\mathcal{T}_.) = 0$. From this it follows that two trees with the same cell distances contain the same observed clones, i.e. we can define a bijection ϕ between the observed clones of $\mathcal{T}_1$ and $\mathcal{T}_2$.

(II) Parent-child relationships between observed clones.
Let i and j be two cells in separate clones of $\mathcal{T}_1$, i.e. $c(i) \neq c(j)$. Then, $c(i) = \text{pa}(c(j))$ if and only if

   (i) $d_{ij}(\mathcal{T}_.) = 1$ and
   (ii) $d_{0j}(\mathcal{T}_.) = d_{0i}(\mathcal{T}_.) + 1$.

Since the pairwise cell distances are equal in both trees, ϕ preserves the parent-child relationships between observed clones.

(III) Assignment of unobserved clones.
Let v_i be an unobserved clone of $\mathcal{T}_1$. Then v_i has an observed descendant $v_j \in T_1$, since all leaf nodes of $\mathcal{T}_1$ contain at least one cell by the definition of the pairwise cell shortest-path distance. We call v_i the n-th ancestor of v_j. Since the distances

from the root to v_j in $\mathcal{T}_1$ and to $\phi(v_j)$ in $\mathcal{T}_2$ are the same, respectively, we can assign $\phi(v_i)$ as the n-th ancestor of $\phi(v_j)$.

This is a well-defined function as the assignment does not depend on the specific choice of the descendant v_j. To see this, choose another observed descendant $\bar{v}_j$. Then v_i is the $\bar{n}$-th ancestor of $\bar{v}_j$. The distances $d_{0v_j}(\mathcal{T}_1)$, $d_{0\bar{v}_j}(\mathcal{T}_1)$ and $d_{v_iv_j}(\mathcal{T}_1)$ uniquely determine the length of the branches connecting the three nodes via v_i. Since those distances are the same in $\mathcal{T}_2$ it follows that the n-th ancestor of $\phi(v_j)$ is the same node as the $\bar{n}$-th ancestor of $\phi(\bar{v}_j)$.

This function defines a bijection between the vertices, since the same construction from $\mathcal{T}_2$ to $\mathcal{T}_1$ defines an inverse.

(IV) General parent-child relationships.
Assuming that $v_i = \text{pa}(v_j)$ we consider the following cases to show that $\phi(v_i) = \text{pa}(\phi(v_j))$:

- (i) v_i and v_j observed – see step (II).
- (ii) $v_i = \text{pa}(v_j)$ and v_i unobserved
  Assign $\phi(v_i)$ following step (III) as first ancestor of $\phi(v_j)$. Then $\phi(v_i) = \text{pa}(\phi(v_j))$.
- (iii) $v_i = \text{pa}(v_j)$ and v_j unobserved
  Choose v_k so that it is an observed descendant of v_j. Assign $\phi(v_j)$ following step (III). Then $\phi(v_j)$ is the n-th ancestor of $\phi(v_k)$ and $\phi(v_i)$ is the $(n+1)$-th ancestor of $\phi(v_k)$, so that $\phi(v_i) = \text{pa}(v_j)$.
- (iv) $v_i = \text{pa}(v_j)$ and both unobserved
  Choose v_k so that it is an observed descendant of v_j. Assign $\phi(v_i)$ and $\phi(v_j)$ following step (III). Then $\phi(v_j)$ is the n-th ancestor of $\phi(v_k)$ and $\phi(v_i)$ is the $(n+1)$-th ancestor of $\phi(v_k)$, so that $\phi(v_i) = \text{pa}(v_j)$.

The same construction from $\mathcal{T}_2$ to $\mathcal{T}_1$ defines the inverse of ϕ. Therefore, $v_i = \text{pa}(v_j) \Leftrightarrow \phi(v_i) = \phi(\text{pa}(v_j))$.

□

With this we can show that $d(\mathcal{T}_1, \mathcal{T}_2)$ satisfies all the requirements of a metric.

Proof that pairwise cell shortest-path distance is a metric

(I) Non-negativity: $d(\mathcal{T}_1, \mathcal{T}_2) \geq 0$
Non-negativity follows from defining the distance as a sum of absolute values.

(II) Coincidence axiom: $d(\mathcal{T}_1, \mathcal{T}_2) = 0$ if and only if $\mathcal{T}_1 = \mathcal{T}_2$

- (i) If $\mathcal{T}_1 = \mathcal{T}_2$, then $d(\mathcal{T}_1, \mathcal{T}_2) = d(\mathcal{T}_1, \mathcal{T}_1) = 0$.
- (ii) If $d(\mathcal{T}_1, \mathcal{T}_2) = 0$, then $\mathcal{T}_1 = \mathcal{T}_2$ as shown in the lemma.

(III) Symmetry: $d(\mathcal{T}_1, \mathcal{T}_2) = d(\mathcal{T}_2, \mathcal{T}_1)$
Symmetry follows from $|d_{ij}(\mathcal{T}_1) - d_{ij}(\mathcal{T}_2)| = |d_{ij}(\mathcal{T}_2) - d_{ij}(\mathcal{T}_1)|$.

(IV) Triangle inequality: $d(\mathcal{T}_1, \mathcal{T}_3) \leq d(\mathcal{T}_1, \mathcal{T}_2) + d(\mathcal{T}_2, \mathcal{T}_3)$
The usual triangle inequality for real numbers implies that

$$|d_{ij}(\mathcal{T}_1) - d_{ij}(\mathcal{T}_3)| \leq |d_{ij}(\mathcal{T}_1) - d_{ij}(\mathcal{T}_2)| + |d_{ij}(\mathcal{T}_2) - d_{ij}(\mathcal{T}_3)|.$$

Hence for the pairwise cell shortest-path distance we find

$$\begin{aligned} d(\mathcal{T}_1, \mathcal{T}_3) &= \sum_{i=0}^{n-1} \sum_{j=i+1}^{n} |d_{ij}(\mathcal{T}_1) - d_{ij}(\mathcal{T}_3)| \\ &\leq \sum_{i=0}^{n-1} \sum_{j=i+1}^{n} |d_{ij}(\mathcal{T}_1) - d_{ij}(\mathcal{T}_2)| + \sum_{i=0}^{n-1} \sum_{j=i+1}^{n} |d_{ij}(\mathcal{T}_2) - d_{ij}(\mathcal{T}_3)| \\ &= d(\mathcal{T}_1, \mathcal{T}_2) + d(\mathcal{T}_2, \mathcal{T}_3). \end{aligned}$$

□

References

[1] Yingrui Li, Xun Xu, Luting Song, Yong Hou, Zesong Li *et al.* Single-cell sequencing analysis characterizes common and cell-lineage-specific mutations in a muscle-invasive bladder cancer. *GigaScience*, 1(1):12, 2012.

[2] Yong Hou, Luting Song, Ping Zhu, Bo Zhang, Ye Tao *et al.* Single-cell exome sequencing and monoclonal evolution of a JAK2-negative myeloproliferative neoplasm. *Cell*, 148(5):873–885, Mar 2012.
