# Supplementary material for: OncoNEM: inferring tumor evolution from single-cell sequencing data
Source: Genome Biol. 2016 Apr 15;17:69. doi: 10.1186/s13059-016-0929-9 (PMC4832472; doi:10.1186/s13059-016-0929-9)
Supplement: Additional file 4 — PDF of compiled knitr script for simulation studies. A PDF file reproducing the results of the simulation studies. (PDF 594 kb) [file 13059_2016_929_MOESM4_ESM.pdf]

Supplement to
OncoNEM: Inferring tumour evolution from single-cell sequencing data
# - Simulation studies -

Edith M Ross and Florian Markowetz

February 29, 2016

Contents

1 Setup **2**
1.1 Short version 2
1.2 Installing the oncoNEM package 2
1.3 Installing other packages used in this vignette 2

2 Robustness of oncoNEM to changes in parameter estimates **3**
2.1 Figure 3 3
2.2 Supplementary Figure 1 6

3 Parameter estimation accuracy and method comparison **11**
3.1 Figure 3B 25
3.2 Figure 5 28

4 Dependence of oncoNEM results on threshold ϵ (Figure 4) **31**

5 Session Info **34**

1 Setup

1.1 Short version

A short version of this script, reproducing only a small subset of the simulation results shown in the paper, can be run by setting `short` to `TRUE` in the following code chunk.

```
short <- FALSE
```

1.2 Installing the oncoNEM package

The oncoNEM package uses functions from the following R packages: Rcpp, igraph, ggm. To install those packages run the following commands in R:

```
install.packages(c('Rcpp','igraph','ggm'))
```

We recommend using a C++ compiler that supports OpenMP (`http://openmp.org`) for installing the oncoNEM package for parallel computing. The oncoNEM package (`https://bitbucket.org/edith_ross/onconem`) can be installed from bitbucket by running the following commands in R

```
install.packages('devtools')
library(devtools)
install_bitbucket('edith_ross/oncoNEM')
```

1.3 Installing other packages used in this vignette

In addition to oncoNEM, this script uses the R packages ggplot2, reshape2, bitphylogenyR, foreach, doParallel, cluster, ape, phangorn and KimAndSimon. Instructions on how to install Bitphylogeny and its dependencies can be found on `bitbucket.org/ke_yuan/bitphylogeny`. Like OncoNEM, KimAndSimon can be installed from bitbucket (`https://bitbucket.org/edith_ross/KimAndSimon`) using devtools:

```
install_bitbucket('edith_ross/KimAndSimon')
```

To install the remaining packages run the following commands in R:

```
install.packages(c('ggplot2','reshape2','foreach','doParallel','cluster','ape','phangorn'))
```

Then we load all the required packages.

```
library(oncoNEM)
library(ggplot2)
library(reshape2)
library(bitphylogenyR)
library(foreach)
library(doParallel)
library(cluster)
library(ape)
library(phangorn)
library(KimAndSimon)
```

Additionally, MrBayes (`http://mrbayes.sourceforge.net/`) needs to be installed.

2 Robustness of oncoNEM to changes in parameter estimates

2.1 Figure 3

To assess the robustness of oncoNEM to changes in parameter estimates, we first simulate a data set.

```
## simulate Data set
set.seed(8)
dat <- simulateData(N.cells = 20,
                    N.clones = 10,
                    N.unobs = 2,
                    N.sites = 200,
                    FPR = 0.2,
                    FNR = 0.1,
                    p.missing = 0.2,
                    randomizeOrder = TRUE)
```

Then we run the oncoNEM inference algorithms over a grid of parameter combinations and assess the inferred solutions in terms of likelihood, pairwise cell shortest path distance to ground truth and V-measure compared to ground truth.

```
if (short) {
  file4Exists <- file.exists('Res/Figure3_short.RData')
} else {
  file4Exists <- file.exists('Res/Figure3.RData')
}
if (!file4Exists) {
  ## define parameter grid
  test.fpr <- test.fnr <- seq(from=0.01,to=0.99,length.out=29)
  if (short) {
    test.fpr <- test.fpr[2:11]
    test.fnr <- test.fnr[2:11]
  }

  ## initialize matrices to store performance results
  dist <- vMes <- llh <- llhClust <- matrix(0,nrow=length(test.fpr),
                                            ncol=length(test.fnr))


  ## run oncoNEM over parameter grid
  for (i.alpha in 1:length(test.fpr)) {
    for (i.beta in 1:length(test.fnr)) {

      ## run oncoNEM
      oNEM <- oncoNEM$new(Data=dat$D,
                          FPR=test.fpr[i.alpha],
                          FNR=test.fnr[i.beta])
      oNEM$search(delta=200)
      # search for hidden nodes
      oNEM.expanded <- expandOncoNEM(oNEM,
                                     delta=100,
                                     epsilon=10,
                                     checkMax=1000,
                                     app=TRUE)
      # cluster
      oncoTree <- clusterOncoNEM(oNEM.expanded,
                                 epsilon=10)
      # relabel cells to make things comparable
```

```
      oncoTree$clones <- relabelCells(clones = oncoTree$clones,
                                      labels = as.numeric(colnames(dat$D)))

      ## measure performance
      dist[i.alpha,i.beta] <- treeDistance(tree1 = dat,tree2=oncoTree)
      vMes[i.alpha,i.beta] <- vMeasure(trueClusters = dat$clones,
                                       predClusters = oncoTree$clones)
      llh[i.alpha,i.beta] <- oNEM$best$llh
      llhClust[i.alpha,i.beta] <- oncoTree$llh

    }
  }
  if (short) {
    save(dist,vMes,llh,llhClust,test.fpr,test.fnr,file='Res/Figure3_short.RData')
  } else {
    save(dist,vMes,llh,llhClust,test.fpr,test.fnr,file='Res/Figure3.RData')
  }
}
```

Next we plot heatmaps of the likelihood landscape, the pairwise cell shortest-path distance and the V-measure.

```
if (short) {
  load('Res/Figure3_short.RData')
} else {
  load('Res/Figure3.RData')
}
## prepare data for ggplot
## - grid
xy <- as.matrix(expand.grid(1:length(test.fpr),1:length(test.fnr)))
## - distance (normalized to highest measured distance to map it onto [0,1])
xy.dist <-  dist[xy]/max(dist)
## - V-Measure
xy.vMes <- vMes[xy]
## - likelihood (convert llh to log(Bayes Factor) relative to highest scoring solution)
xy.lBF <- llhClust[xy]-max(llhClust)
## define x and y axis
x <- test.fpr[xy[,1]]
y <- test.fnr[xy[,2]]


## find best parameter combination based on results from initial search
indx <- which(llh==max(llh),arr.ind=TRUE)
fpr.est <- test.fpr[indx[1]]
fnr.est <- test.fnr[indx[2]]

errorRate <- data.frame(x=c(dat$FPR,fpr.est),
                        y=c(dat$FNR,fnr.est),
                        Parameter=c('Ground truth','Estimate'))

## plot log(Bayes factor)
df <- data.frame(x=x,
                 y=y,
                 lBF=xy.lBF,
                 stringsAsFactors = FALSE)
df <- melt(df,id=c('x','y'),variable.name = "Measure")
```

```
ggplot(df, aes(x, y)) +
  geom_raster(aes(fill = value),
              hjust = 0.5,
              vjust = 0.5)+
  labs(x = 'False positive rate',
       y = 'False negative rate') +
  facet_grid(.~Measure)+
  scale_fill_gradientn(colours = rainbow(7),
                       name='log(Bayes factor)',
                       limits=c(-1000,0)) +
  geom_point(data=errorRate,
             aes(x=x,y=y,shape=Parameter),
             colour="black",
             bg="white",
             size=4) +
  scale_shape_manual(values = c('Ground truth' = 24, 'Estimate' = 25)) +
  theme(axis.text  = element_text(size=12),
        axis.text.x =  element_text(angle = 45, hjust=1),
        axis.title = element_text(size=16),
        axis.title.y = element_text(vjust = 1),
        strip.text.x = element_text(size=16),
        legend.text = element_text(size=12),
        aspect.ratio = 1)
```

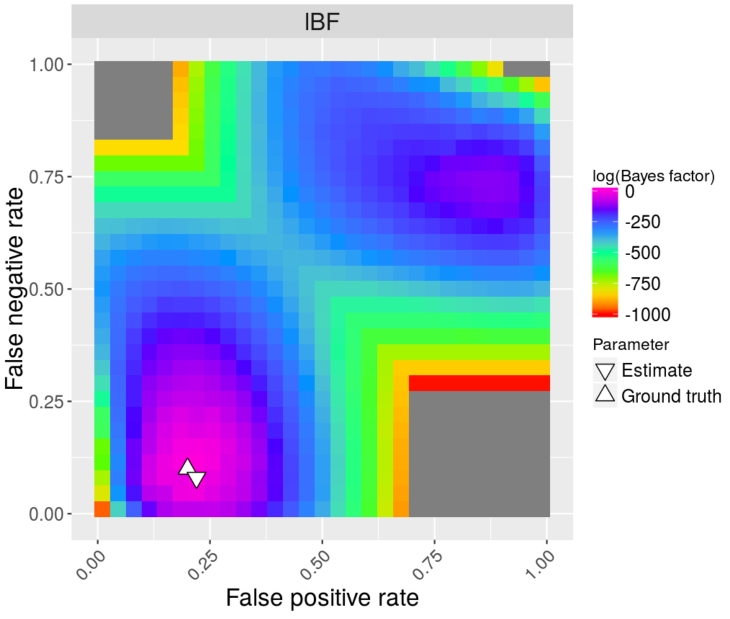


```
## plot V-measure and Distance
df <- data.frame(x=x,
                 y=y,
                 Distance=xy.dist,
                 vMeasure=xy.vMes,
                 stringsAsFactors = FALSE)
df <- melt(df,id=c('x','y'),variable.name = "Measure")
```

```
ggplot(df, aes(x, y)) +
  geom_raster(aes(fill = value)) +
  labs(x = 'False positive rate',
       y = 'False negative rate') +
  facet_grid(.~Measure)+
  scale_fill_gradientn(colours = rainbow(7),
                       name='Distance/V-measure',
                       limits=c(0,1)) +
  geom_point(data=errorRate,
             aes(x=x,y=y,shape=Parameter),
             colour="black",
             bg="white",
             size=4) +
  scale_shape_manual(values = c('Ground truth' = 24, 'Estimate' = 25)) +
  theme(axis.text  = element_text(size=12),
        axis.text.x =  element_text(angle = 45, hjust=1),
        axis.title = element_text(size=16),
        axis.title.y = element_text(vjust = 1),
        strip.text.x = element_text(size=16),
        legend.text = element_text(size=12),
        aspect.ratio = 1)
```

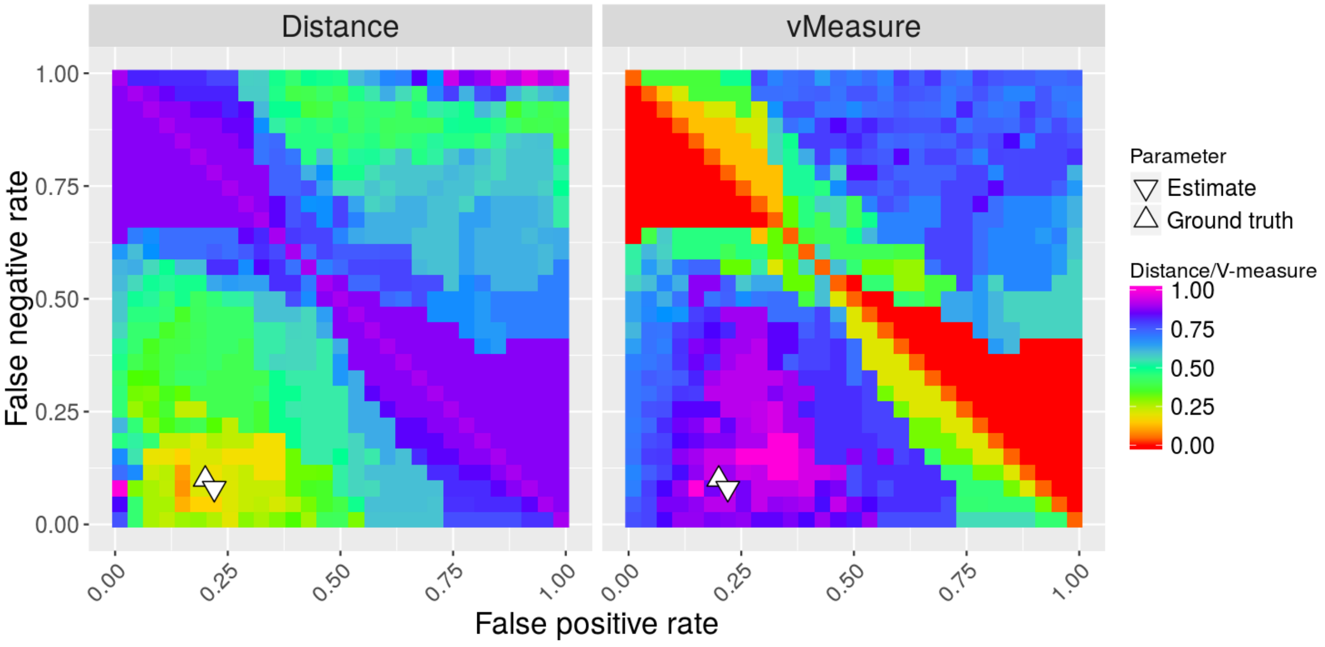

The estimated FPR is 0.22 (true value is 0.2), the estimated FNR is 0.08 (true value is 0.1).

2.2 Supplementary Figure 1

Next, we test if these results can be reproduced on a data set with very low false positive rates (10^{-5}). We start again by simulating a data set, run the inference and plot the results.

```
## simulate Data set
set.seed(8)
dat <- simulateData(N.cells = 20,
                    N.clones = 10,
                    N.unobs = 2,
                    N.sites = 200,
```

```
                    FPR = 10^(-5),
                    FNR = 0.1,
                    p.missing = 0.2,
                    randomizeOrder = TRUE)
```

Then we run the oncoNEM inference algorithms over a grid of parameter combinations and assess the inferred solutions in terms of likelihood, pairwise cell shortest path distance to ground truth and V-measure compared to ground truth.

```
if (short) {
  file4Exists <- file.exists('Res/Figure1Supp_short.RData')
} else {
  file4Exists <- file.exists('Res/Figure1Supp.RData')
}
if (!file4Exists) {
  ## define parameter grid
  test.fpr <- test.fnr <- seq(from=0.01,to=0.99,length.out=29)
  if (short) {
    test.fpr <- test.fpr[2:11]
    test.fnr <- test.fnr[2:11]
  }

  ## initialize matrices to store performance results
  dist <- vMes <- llh <- llhClust <- matrix(0,nrow=length(test.fpr),
                                            ncol=length(test.fnr))


  ## run oncoNEM over parameter grid
  for (i.alpha in 1:length(test.fpr)) {
    for (i.beta in 1:length(test.fnr)) {

      ## run oncoNEM
      oNEM <- oncoNEM$new(Data=dat$D,
                          FPR=test.fpr[i.alpha],
                          FNR=test.fnr[i.beta])
      oNEM$search(delta=200)
      # search for hidden nodes
      oNEM.expanded <- expandOncoNEM(oNEM,
                                     delta=100,
                                     epsilon=10,
                                     checkMax=1000,
                                     app=TRUE)
      # cluster
      oncoTree <- clusterOncoNEM(oNEM.expanded,
                                 epsilon=10)
      # relabel cells to make things comparable
      oncoTree$clones <- relabelCells(clones = oncoTree$clones,
                                      labels = as.numeric(colnames(dat$D)))

      ## measure performance
      dist[i.alpha,i.beta] <- treeDistance(tree1 = dat,tree2=oncoTree)
      vMes[i.alpha,i.beta] <- vMeasure(trueClusters = dat$clones,
                                       predClusters = oncoTree$clones)
      llh[i.alpha,i.beta] <- oNEM$best$llh
      llhClust[i.alpha,i.beta] <- oncoTree$llh

    }
```

```
  }
  if (short) {
    save(dist,vMes,llh,llhClust,test.fpr,test.fnr,file='Res/Figure1Supp_short.RData')
  } else {
    save(dist,vMes,llh,llhClust,test.fpr,test.fnr,file='Res/Figure1Supp.RData')
  }
}
```

```
if (short) {
  load('Res/Figure1Supp_short.RData')
} else {
  load('Res/Figure1Supp.RData')
}
## prepare data for ggplot
## - grid
xy <- as.matrix(expand.grid(1:length(test.fpr),1:length(test.fnr)))
## - distance (normalized to highest measured distance to map it onto [0,1])
xy.dist <-  dist[xy]/max(dist)
## - V-Measure
xy.vMes <- vMes[xy]
## - likelihood (convert llh to log(Bayes Factor) relative to highest scoring solution)
xy.lBF <- llhClust[xy]-max(llhClust)
## define x and y axis
x <- test.fpr[xy[,1]]
y <- test.fnr[xy[,2]]


## find best parameter combination based on results from initial search
indx <- which(llh==max(llh),arr.ind=TRUE)
fpr.est <- test.fpr[indx[1]]
fnr.est <- test.fnr[indx[2]]

errorRate <- data.frame(x=c(dat$FPR,fpr.est),
                        y=c(dat$FNR,fnr.est),
                        Parameter=c('Ground truth','Estimate'))

## plot log(Bayes factor)
df <- data.frame(x=x,
                 y=y,
                 lBF=xy.lBF,
                 stringsAsFactors = FALSE)
df <- melt(df,id=c('x','y'),variable.name = "Measure")

ggplot(df, aes(x, y)) +
  geom_raster(aes(fill = value),
              hjust = 0.5,
              vjust = 0.5)+
  labs(x = 'False positive rate',
       y = 'False negative rate') +
  facet_grid(.~Measure)+
  scale_fill_gradientn(colours = rainbow(7),
                       name='log(Bayes factor)',
                       limits=c(-1000,0)) +
  geom_point(data=errorRate,
             aes(x=x,y=y,shape=Parameter),
```

```
             colour="black",
             bg="white",
             size=4) +
  scale_shape_manual(values = c('Ground truth' = 24, 'Estimate' = 25)) +
  theme(axis.text  = element_text(size=12),
        axis.text.x =  element_text(angle = 45, hjust=1),
        axis.title = element_text(size=16),
        axis.title.y = element_text(vjust = 1),
        strip.text.x = element_text(size=16),
        legend.text = element_text(size=12),
        aspect.ratio = 1)
```

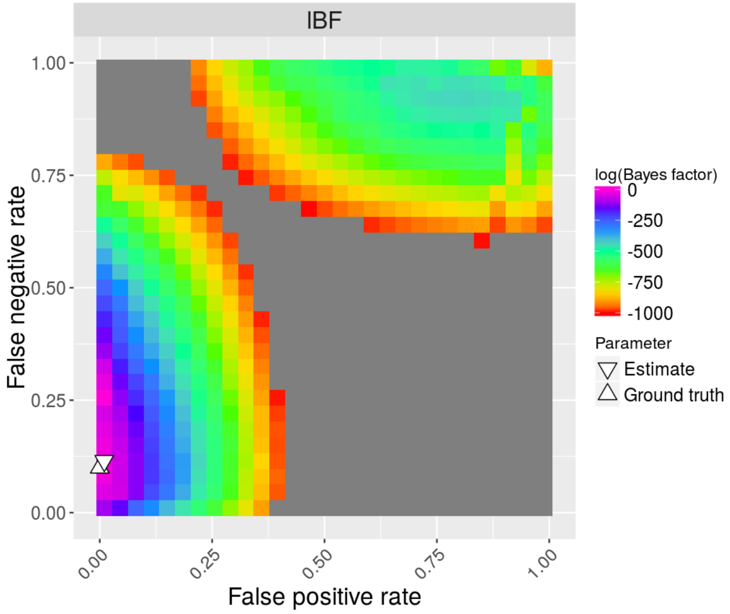


```
## plot V-measure and Distance
df <- data.frame(x=x,
                 y=y,
                 Distance=xy.dist,
                 vMeasure=xy.vMes,
                 stringsAsFactors = FALSE)
df <- melt(df,id=c('x','y'),variable.name = "Measure")

ggplot(df, aes(x, y)) +
  geom_raster(aes(fill = value)) +
  labs(x = 'False positive rate',
       y = 'False negative rate') +
  facet_grid(.~Measure)+
  scale_fill_gradientn(colours = rainbow(7),
                       name='Distance/V-measure',
                       limits=c(0,1)) +
  geom_point(data=errorRate,
             aes(x=x,y=y,shape=Parameter),
             colour="black",
             bg="white",
```

```r
             size=4) +
  scale_shape_manual(values = c('Ground truth' = 24, 'Estimate' = 25)) +
  theme(axis.text  = element_text(size=12),
        axis.text.x =  element_text(angle = 45, hjust=1),
        axis.title = element_text(size=16),
        axis.title.y = element_text(vjust = 1),
        strip.text.x = element_text(size=16),
        legend.text = element_text(size=12),
        aspect.ratio = 1)
```

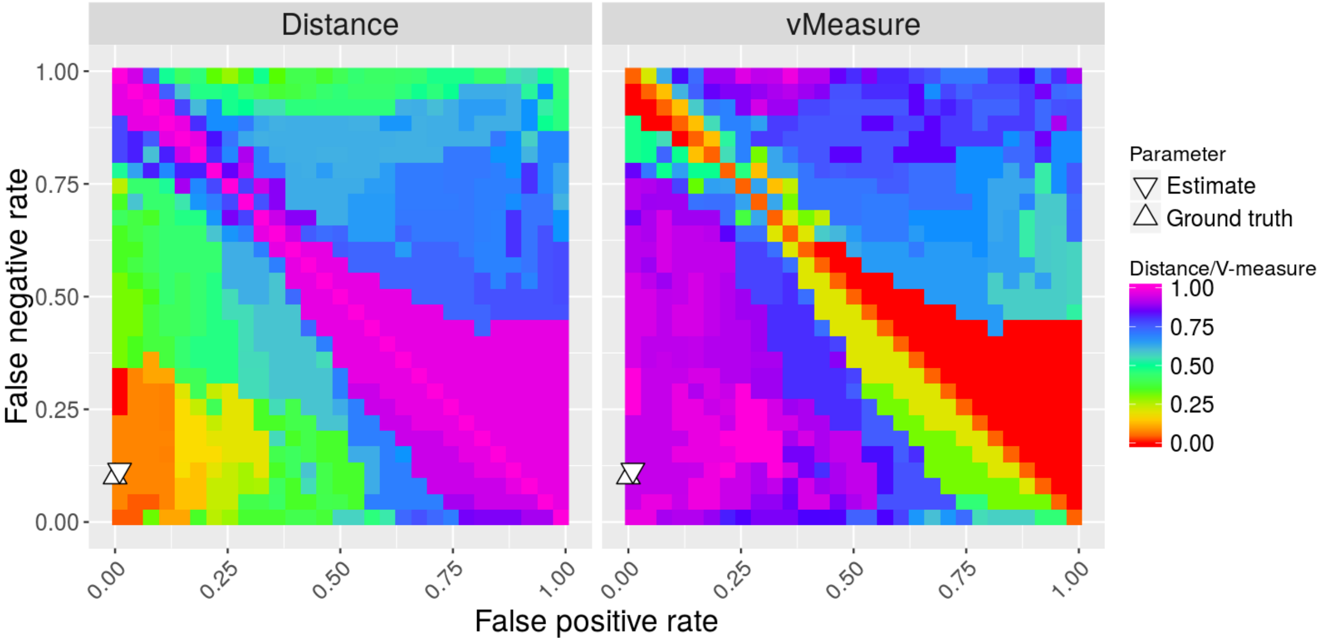

The estimated FPR is 0.01 (true value is 10^{-5}), the estimated FNR is 0.115 (true value is 0.1). Note that the lowest false positive rate used for the parameter estimation was 0.01.

3 Parameter estimation accuracy and method comparison

We start by defining the default parameters and the variable parameters for our method comparison study:

```
## default parameters
n.sites <- 200   ## Number of mutation sites
n.clones <- 10   ## Number of clones
n.cells <- 20    ## Number of sequenced cells
n.unobs <- 0     ## Number of clones in tree that are unobserved
p.missing <- 0.2 ## Fraction of missing values
fpr <- 0.2       ## False positive rate
fnr <- 0.1       ## False negative rate
n.rep <- 5       ## Number of replicates

## simulation parameters that are varied
vary <- c("FNR","FPR","sites","clones","unobs","cells","mis")

## vary FNR
v.FNR <- c(0.05,0.1,0.2,0.3)

## vary FPR
v.FPR <- c(0.05,0.1,0.2,0.3)
## additional simulation parameter
FPRlow <- 10^-5

## vary N sites
v.sites <- c(50,100,200,300)

## vary N clones
v.clones <- c(1,5,10,20)

## vary N unobserved
v.unobs <- c(1,2,3,4)

## vary N cells per clone
v.cells <- c(1,2,3,5)*n.clones

## vary fraction of missing values
v.mis <- c(0.1,0.2,0.3,0.4)

dir.create('SimData',showWarnings = FALSE)
save(list = c('n.sites','n.clones','n.cells','n.unobs','n.rep','p.missing',
              'fpr','fnr','v.FNR','v.FPR','v.sites','v.clones','v.unobs',
              'v.cells','v.mis','vary','FPRlow'),
     file='SimData/params.RData')
```

Next we simulate 5 data sets for each varied simulation parameter.

```
set.seed(7)

## vary FNR
for (i.FNR in 1:length(v.FNR)) {
  for (i.rep in 1:n.rep) {
    dat <- simulateData(N.cells = n.cells,
                        N.clones = n.clones,
                        N.unobs = n.unobs,
                        N.sites = n.sites,
```

```
                         FPR = fpr,
                         FNR = v.FNR[i.FNR],
                         p.missing = p.missing,
                         randomizeOrder = TRUE)
    ## add normal cell to root
    dat$D <- cbind('0'=rep(0,nrow(dat$D)),dat$D)
    dat$clones[[1]] <- 0
    ## save
    save(dat,file=paste0('SimData/v_FNR-',v.FNR[i.FNR],'_rep',i.rep,'.RData'))
  }
}

## vary FPR
for (i.FPR in 1:length(v.FPR)) {
  for (i.rep in 1:n.rep) {
    dat <- simulateData(N.cells = n.cells,
                         N.clones = n.clones,
                         N.unobs = n.unobs,
                         N.sites = n.sites,
                         FPR = v.FPR[i.FPR],
                         FNR = fnr,
                         p.missing = p.missing,
                         randomizeOrder = TRUE)
    ## add normal cell to root
    dat$D <- cbind('0'=rep(0,nrow(dat$D)),dat$D)
    dat$clones[[1]] <- 0
    ## save
    save(dat,file=paste0('SimData/v_FPR-',v.FPR[i.FPR],'_rep',i.rep,'.RData'))
  }
}

## vary N sites
for (i.sites in 1:length(v.sites)) {
  for (i.rep in 1:n.rep) {
    dat <- simulateData(N.cells = n.cells,
                         N.clones = n.clones,
                         N.unobs = n.unobs,
                         N.sites = v.sites[i.sites],
                         FPR = fpr,
                         FNR = fnr,
                         p.missing = p.missing,
                         randomizeOrder = TRUE)
    ## add normal cell to root
    dat$D <- cbind('0'=rep(0,nrow(dat$D)),dat$D)
    dat$clones[[1]] <- 0
    ## save
    save(dat,file=paste0('SimData/v_sites-',v.sites[i.sites],'_rep',i.rep,'.RData'))
  }
}

## vary N clones
for (i.clones in 1:length(v.clones)) {
  for (i.rep in 1:n.rep) {
    dat <- simulateData(N.cells = n.cells,
                         N.clones = v.clones[i.clones],
                         N.unobs = n.unobs,
```

```
                        N.sites = n.sites,
                        FPR = fpr,
                        FNR = fnr,
                        p.missing = p.missing,
                        randomizeOrder = TRUE)
    ## add normal cell to root
    dat$D <- cbind('0'=rep(0,nrow(dat$D)),dat$D)
    dat$clones[[1]] <- 0
    ## save
    save(dat,file=paste0('SimData/v_clones-',v.clones[i.clones],'_rep',i.rep,'.RData'))
  }
}

## vary N unobserved
for (i.unobs in 1:length(v.unobs)) {
  for (i.rep in 1:n.rep) {
    dat <- simulateData(N.cells = n.cells,
                        N.clones = n.clones,
                        N.unobs = v.unobs[i.unobs],
                        N.sites = n.sites,
                        FPR = fpr,
                        FNR = fnr,
                        p.missing = p.missing,
                        randomizeOrder = TRUE)
    ## add normal cell to root
    dat$D <- cbind('0'=rep(0,nrow(dat$D)),dat$D)
    dat$clones[[1]] <- 0
    ## save
    save(dat,file=paste0('SimData/v_unobs-',v.unobs[i.unobs],'_rep',i.rep,'.RData'))
  }
}

## vary N cells per clone
for (i.cells in 1:length(v.cells)) {
  for (i.rep in 1:n.rep) {
    dat <- simulateData(N.cells = v.cells[i.cells],
                        N.clones = n.clones,
                        N.unobs = n.unobs,
                        N.sites = n.sites,
                        FPR = fpr,
                        FNR = fnr,
                        p.missing = p.missing,
                        randomizeOrder = TRUE)
    ## add normal cell to root
    dat$D <- cbind('0'=rep(0,nrow(dat$D)),dat$D)
    dat$clones[[1]] <- 0
    ## save
    save(dat,file=paste0('SimData/v_cells-',v.cells[i.cells],'_rep',i.rep,'.RData'))
  }
}

for (i.mis in 1:length(v.mis)) {
  for (i.rep in 1:n.rep) {
    dat <- simulateData(N.cells = n.cells,
                        N.clones = n.clones,
                        N.unobs = n.unobs,
```

```
                        N.sites = n.sites,
                        FPR = fpr,
                        FNR = fnr,
                        p.missing = v.mis[i.mis],
                        randomizeOrder = TRUE)
    ## add normal cell to root
    dat$D <- cbind('0'=rep(0,nrow(dat$D)),dat$D)
    dat$clones[[1]] <- 0
    ## save
    save(dat,file=paste0('SimData/v_mis-',v.mis[i.mis],'_rep',i.rep,'.RData'))
  }
}
```

In addition we simulate data sets with extremely low numbers of false positive.

```
for (i.rep in 1:n.rep) {
    dat <- simulateData(N.cells = n.cells,
                        N.clones = n.clones,
                        N.unobs = n.unobs,
                        N.sites = n.sites,
                        FPR = FPRlow,
                        FNR = fnr,
                        p.missing = p.missing,
                        randomizeOrder = TRUE)
    ## add normal cell to root
    dat$D <- cbind('0'=rep(0,nrow(dat$D)),dat$D)
    dat$clones[[1]] <- 0
    ## save
    save(dat,file=paste0('SimData/v_FPR-',FPRlow,'_rep',i.rep,'.RData'))
}
v.FPR <- c(FPRlow,v.FPR)
```

Then we infer trees using oncoNEM and the three baseline methods. For the short version of this script, only one replicate and one setting per simulation parameter is used.

```
if (short) {
 n.rep <- 1
 for (varyX in vary) {
   v.X <- get(paste0('v.',varyX))
   assign(paste0('v.',varyX),v.X[3])
 }
}
```

We start by running oncoNEM with and without parameter estimation.

```
dir.create('Res',showWarnings = FALSE)
dir.create('Res/MethodComparison',showWarnings = FALSE)

## vary FNR
## vary FPR
## vary N sites
## vary N clones
## vary N unobserved
## vary N cells per clone
## vary fraction of missing values
```

```r
for (varyX in vary) {
  v.X <- get(paste0('v.',varyX))

  for (i.vary in 1:length(v.X)) {

    for (i.rep in 1:n.rep) {

      if (!file.exists(paste0('Res/MethodComparison/oncoNEM_result_v_',varyX,
                              '-',v.X[i.vary],'_rep',i.rep,'.RData'))) {

        ## load Data
        load(paste0('SimData/v_',varyX,'-',v.X[i.vary],'_rep',i.rep,'.RData'))

        ## ---------------------------------------------------------------------
        ## estimate Parameters
        test.fpr <- test.fnr <- seq(from=0.01,to=0.5,length.out=15)
        llh <- matrix(0,nrow=length(test.fpr),ncol=length(test.fnr))
        for (i.fpr in 1:length(test.fpr)) {
          for (i.fnr  in 1:length(test.fnr)) {
            ## initialize oncoNEM
            oNEM <- oncoNEM$new(Data=dat$D,
                                FPR=test.fpr[i.fpr],
                                FNR=test.fnr[i.fnr])
            ## Run search algorithm until best tree has not changed for at least
            ## delta steps.
            oNEM$search(delta=200)
            llh[i.fpr,i.fnr] <- oNEM$best$llh
          }
        }
        rm(oNEM)

        ## find best parameter combination
        indx <- which(llh==max(llh),arr.ind=TRUE)
        fpr.est <- test.fpr[indx[1]]
        fnr.est <- test.fnr[indx[2]]

        ## ---------------------------------------------------------------------
        ## Run oncoNEM with estimated Parameters
        ## initialize oncoNEM
        oNEM.est <- oncoNEM$new(Data=dat$D,
                                FPR=fpr.est,
                                FNR=fnr.est)
        ## Run search algorithm until best tree has not changed for at least
        ## delta steps.
        oNEM.est$search(delta=200)

        # search for hidden nodes
        oNEM.est.expanded <- expandOncoNEM(oNEM.est,epsilon = 10,delta = 200)
        rm(oNEM.est)

        # cluster
        oncoTree.est <- clusterOncoNEM(oNEM.est.expanded, epsilon=10)
        rm(oNEM.est.expanded)

        # relabel cells from column indices to cell indices as in ground truth
        # tree
```

```
        oncoTree.est$clones <- relabelCells(clones = oncoTree.est$clones,
                                            labels = as.numeric(colnames(dat$D)))


        ## ----------------------------------------------------------------------
        ## As comparison run oncoNEM with true parameters
        ## initialize oncoNEM
        oNEM <- oncoNEM$new(Data=dat$D,
                            FPR=dat$FPR.effective,
                            FNR=dat$FNR)
        ## Run search algorithm
        oNEM$search(delta=200)

        # search for hidden nodes
        oNEM.expanded <- expandOncoNEM(oNEM,epsilon = 10,delta=200)
        rm(oNEM)

        # cluster
        oncoTree <- clusterOncoNEM(oNEM.expanded, epsilon=10)
        rm(oNEM.expanded)

        # relabel cells from column indices to cell indices
        oncoTree$clones <- relabelCells(clones = oncoTree$clones,
                                        labels = as.numeric(colnames(dat$D)))

        ## ----------------------------------------------------------------------
        ## save inference results so far
        ssave(c('llh','fpr.est','fnr.est','oncoTree.est','oncoTree'),
              file=paste0('Res/MethodComparison/oncoNEM_result_v_',varyX,
                          '-',v.X[i.vary],'_rep',i.rep,'.RData'))
      }
    }
  }
}
```

Next, we run the BitPhylogeny inference.

```
dir.create('Res/BitPhylo_Output/',recursive = TRUE,showWarnings = FALSE)
#setup parallel backend to use 8 processors
cl<-makeCluster(4)
registerDoParallel(cl)

for (varyX in vary) {
  v.X <- get(paste0('v.',varyX))

  resultsExist <- sapply(paste0(rep(paste0('Res/BitPhylo_Output/v_',varyX,'-',v.X),each=n.rep),'_rep',
                                1:n.rep,'.csv/treescripts/tree-freq.csv'),
                         file.exists)

  if (!all(resultsExist)) {
    #number of iterations
    iters<-n.rep*length(v.X)

    #loop
    ls<-foreach(i=1:iters) %dopar% {
      library("bitphylogenyR")
```

```
      i.X <- ceiling(i/n.rep)
      i.rep <- i-(i.X-1)*n.rep

      load(paste0('SimData/v_',varyX,'-',v.X[i.X],'_rep',i.rep,'.RData'))
      # save data to file -> must be cells x sites
      write.csv(t(dat$D[,order(as.numeric(colnames(dat$D)))]),
                file=paste0('SimData/v_',varyX,'-',v.X[i.X],'_rep',i.rep,'.csv'),
                row.names=FALSE)
      # run bitphyloR
      bitphyloR(fin = paste0('SimData/v_',varyX,'-',v.X[i.X],'_rep',i.rep,'.csv'),
                fout='Res/BitPhylo_Output/',
                contains_true_label=FALSE,
                n=50000,
                b=30000,
                t=5,
                mode='mutation')

    }
  }
}

stopCluster(cl)
```

To be able evaluate the performance measures for BitPhylogeny later in this script, we define a function that identifies the best BitPhylogeny tree.

```
getBitPhyloTree <- function(dir) {
  ## Choose unique node num for which freq is highest.
  treeFreq <- read.csv(file = paste0(dir,'/treescripts/tree-freq.csv'))
  numNodes <- treeFreq$unique_node_num[which.max(treeFreq$freq)]

  ## read in corresponding tree
  g <- read.graph(file = paste0(dir,'/treescripts/nodes-',numNodes,'.graphml'),
                  format =  "graphml")
  clones <- lapply((strsplit(V(g)$members,split = " ")),function(x) as.numeric(x))

  V(g)$name <- 1:vcount(g)
  return(list(g=g,clones=clones))
}
```

Before we run the other four baseline methods, we define functions for (i) likelihood optimization of neighbor-joining trees as done by Hughes et al (2014) and (ii) Bayesian phylogenetic inference using MrBayes as done by Eirew et al (2015). Hierarchical clustering and k-means clustering combined with minimum-spanning tree reconstruction are both implemented in the Bitphylogeny R package.

```
## (i) likelihood optimization of NJ tree

bin_to_char <- function(x,seed=7) {
  ## Convert a binary genotype matrix to a character matrix, by randomly assigning
  ## reference and mutant alleles to each site.
  ## Missing values are encoded using the IUPAC ambiguity code
  ## input: x - binary genotype matrix where each column corresponds to an SNV and
  ##            each row corresponds to a cell
  ## output: character genotype matrix where each SNV has now a randomly assigned
  ##         wt allele and mutant allele
```

```
  set.seed(seed)
  ## IUPAC ambiguity code for two letters
  ambigCode <- c(m='ac',m='ca',r='ag',r='ga',w='at',w='ta',s='cg',s='gc',
                 y='ct',y='tc',k='gt',k='tg')

  ## randomly assign wildtype+ref allele to convert 1/0 matrix to characters
  for (i in 1:ncol(x)) {
    gtyp <- sample(x = c('a','c','g','t'), size=2, replace = FALSE)
    x[x[,i]==0,i] <- gtyp[1] ## wt genotype
    x[x[,i]==1,i] <- gtyp[2] ## mutant genotype
    x[x[,i]==2,i] <- names(ambigCode)[paste(gtyp,collapse="")==ambigCode] # ambig genotype
  }

  return(x)
}

clusterPhylo <- function(phylo,K) {
  ## Infer cell clusters in the phylogenetic tree by hierarchical clustering of the distance matrix

  ## convert tree from class phylo to class igraph
  g <- graph.edgelist(el=phylo$edge)
  E(g)$weight <- phylo$edge.length
  ## extract distance matrix for cells
  dm <- shortest.paths(g)
  rownames(dm) <- colnames(dm) <- V(g)
  dis <- as.dist(dm[as.character(1:nrow(x)),as.character(1:nrow(x))])
  ## apply hierarchical clustering to the distance matrix of the optimized NJtree to infer clusters
  hc_cand <- lapply(K, function(ii) cutree(hclust(dis), ii))
  hc_silhouette_res <- sapply(1:length(K), function(ii) summary(silhouette(hc_cand[[ii]],
                                                                            dis))$avg.width)
  ## choose number of clusters that maximize silhouette score
  idx <- which.max(hc_silhouette_res)
  hc_label <- hc_cand[[idx]]

  ## return cluster labels
  return(hc_label)
}

## Infers cluster labels for each cell using likelihood optimization of a neighbour joining tree.
get_label_lo <- function (x,K=2:(nrow(x)-1)) {
  ## input: x - binary genotype matrix where each row corresponds to an SNV and
  ##            each column corresponds to a cell
  ## output: vector containing the cluster label for each cell

  ## convert binary genotype matrix to character matrix by randomly assigning reference and mutant allele
  xChar <- bin_to_char(x)

  ## Step 1: Build NJ tree
  ## Convert format of genotype matrix for dist.dna function
  x.DNAbin <- as.DNAbin(xChar)
  ## Calculated pairwise distances between cells using a generalized Kimura model
  dd <- dist.dna(x.DNAbin,model="K81",pairwise.deletion = TRUE)
  if (any(is.infinite(dd)|is.nan(dd))) { ## test if distance calculation failed for any entries
    return(NA) ## NaN/Inf cause error in likelihood optimization
  }
```

```r
  ## infer tree using modified neighbour-joining algorithm
  NJTree <- bionjs(X = dd)

  ## Step 2: Perform ML-optimization
  ## Convert format of genotype matrix for likelihood optimization
  pD <- phyDat(xChar,type="DNA")
  ## compute likelihood of NJTree given genotypes
  suppressWarnings(x.pml <- pml(NJTree,data=pD,model='GTR'))
  ## Perform likelihood optimization under a generalized time reversible (GTR) substitution model
  x.optPml <- optim.pml(x.pml,model='GTR')

  ## Step 3: Infer clusters from estimated tree
  lo_label <- clusterPhylo(x.optPml$tree,K=K)

  return(lo_label)
}

## (ii) Bayesian phylogenetic inference using MrBayes
writeNexus <- function(D,fileNameBase) {
  ## D - columns correspond to species, rows correspond to sites
  D <- apply(D,1,as.character)
  ## code ambiguous sites
  if (any(is.na(D))) {
    D[is.na(D)] <- '{0,1}'
  } else if (any(D==2)) {
    D[D=='2'] <- '{0,1}'
  }
  colnames(D) <- 1:ncol(D)
  ## write to file in nexus format
  write.nexus.data(x=D,file=paste0(fileNameBase,'.nex'))
  ## change data type from DNA to Restriction
  system(command = paste0("awk '{ gsub(\"DNA\",\"Restriction\"); print $0 }' ",
                          fileNameBase,".nex > ",fileNameBase,"2.nex"))
  ## rename file
  system(command = paste0("mv ",fileNameBase,"2.nex ", fileNameBase,".nex"))
}

MrBayes <- function(fileNameBase,ngen,verbose=FALSE,seeds=sample(100,2)) {
  ## write MrBayes script
  fileConn <- file(paste0(fileNameBase,"MrBayes.txt"))
  writeLines(c("set autoclose=yes nowarn=yes;",
               paste0("set seed=",seeds[1]," swapseed=",seeds[2],";"),
               ## load data
               paste0("execute ",fileNameBase,".nex;"),
               ## set parameters
               "lset rates=equal coding=all;",
               "prset statefreqpr=Dirichlet(1,1);",
               ## run inference
               paste0("mcmc nruns=2 ngen=",format(ngen,scientific=FALSE)," burninfrac=0.5;"),
               "sump;",
               "sumt;",
               "quit;"), fileConn)
  close(fileConn)

  ## run MrBayes
  if (verbose) {
```

```
    system(command = paste0("mpirun -np 4 mb ", fileNameBase,"MrBayes.txt"))
  } else {
    system(command = paste0("mpirun -np 4 mb ", fileNameBase, "MrBayes.txt"),ignore.stdout=TRUE)
  }

  ## read consensus tree from MrBayes output file
  ct <- read.nexus(paste0(fileNameBase,".nex.con.tre"))

  ## return consensus tree
  return(ct)
}

## run Bayesian phylogenetic inference
get_label_mb <- function(x,fileNameBase,ngen,K=2:(nrow(x)-1),seeds=sample(100,2)) {

  writeNexus(t(x),fileNameBase=fileNameBase)
  ct <- MrBayes(fileNameBase,ngen=ngen,seeds=seeds)
  MrBayes_labels <- clusterPhylo(ct,K=K)

  return(MrBayes_labels)
}
```

We run the analysis for four of the five the remaining four baseline methods and evaluate the oncoNEM and BitPhylogeny output in terms of V-measure and pairwise-cell shortest path distance.

```
dir.create('Res/MrBayes',showWarnings = FALSE)
set.seed(1)
for (varyX in vary) {
  v.X <- get(paste0('v.',varyX))

  ## initialize list to store results
  RES <- vector("list")

  for (i.vary in 1:length(v.X)) {

    ## initialize matrices to store results
    vMes <- dist <- matrix(0,nrow=n.rep,ncol=7)
    colnames(vMes) <- colnames(dist) <- c("oncoNEM.est","oncoNEM",
                                          "hc","kc","lo","mb","bitPhylogeny")

    for (i.rep in 1:n.rep) {
      ## load Data
      load(paste0('SimData/v_',varyX,'-',v.X[i.vary],'_rep',i.rep,'.RData'))

      ## ----------------------------------------------------------------------
      ## Run baseline methods hc and kc + MST, likelihood optimization of NJ tree (lo),
      ## and Bayesian Inference using MrBayes (mb)

      ## reformat Data for other methods
      Data <- dat$D
      x <- t(Data[,order(as.numeric(colnames(Data)))])

      ## HIERARCHICAL CLUSTERING
      hc <- get_label_hc(x,K=2:(nrow(x)-1))
      hcTree <- list(g=get_mst(hc$genotype),clones=split(as.numeric(names(hc$label)),
                                                         f=hc$label))
```

```r
## K-CENTROIDS
kc <- get_label_kc(x,K=2:(nrow(x)-1))
kcTree <- list(g=get_mst(kc$genotype),clones=split(as.numeric(names(kc$label)),
                                                   f=kc$label))

## likelihood optimization
lo <- get_label_lo(x,K=2:(nrow(x)-1))
if (all(!is.na(lo))) {
  lo_clones <- split(0:(length(lo)-1),f=lo)
} else {
  lo_clones <- NULL
}

## Bayesian phylogenetic inference with MrBayes
if (file.exists(paste0('Res/MrBayes/v_',varyX,'-',v.X[i.vary],'_rep',i.rep,'.nex.con.tre'))) {
  ct <- read.nexus(paste0('Res/MrBayes/v_',varyX,'-',v.X[i.vary],'_rep',i.rep,'.nex.con.tre'))
  mb <- clusterPhylo(ct,K=2:(nrow(x)-1))
} else {
  mb <- get_label_mb(x,fileNameBase=
                       paste0('Res/MrBayes/v_',varyX,'-',v.X[i.vary],'_rep',i.rep),
                     ngen=10^7,
                     K=2:(nrow(x)-1))
}
mb_clones <- split(0:(length(mb)-1),f=mb)

## ----------------------------------------------------------------------
## load oncoNEM results
load(paste0('Res/MethodComparison/oncoNEM_result_v_',varyX,'-',v.X[i.vary],
            '_rep',i.rep,'.RData'))

## ----------------------------------------------------------------------
## load BitPhylogeny results
if (file.exists(paste0('Res/BitPhylo_Output/v_',varyX,'-',v.X[i.vary],
                                 '_rep',i.rep,'.csv'))) {
  # extract BITPHYLOGENY results
  bitTree <-
    getBitPhyloTree(dir = paste0('Res/BitPhylo_Output/v_',varyX,'-',v.X[i.vary],
                                 '_rep',i.rep,'.csv'))
} else {
  bitTree <- NULL
}


## ----------------------------------------------------------------------
## summarize results
# vMeasure (all baseline methods)
vMes[i.rep,"hc"] <- vMeasure(trueClusters = dat$clones,
                             predClusters = hcTree$clones)
vMes[i.rep,"kc"] <- vMeasure(trueClusters = dat$clones,
                             predClusters = kcTree$clones)
if (is.null(lo_clones)) { ## if lo failed
  vMes[i.rep,"lo"] <- NA
} else {
  vMes[i.rep,"lo"] <- vMeasure(trueClusters = dat$clones,
                               predClusters = lo_clones)
```

```
      }
      vMes[i.rep,"mb"] <- vMeasure(trueClusters = dat$clones,
                                   predClusters = mb_clones)
      vMes[i.rep,"oncoNEM.est"] <- vMeasure(trueClusters = dat$clones,
                                            predClusters = oncoTree.est$clones)
      vMes[i.rep,"oncoNEM"] <- vMeasure(trueClusters = dat$clones,
                                        predClusters = oncoTree$clones)
      vMes[i.rep,"bitPhylogeny"] <- vMeasure(trueClusters = dat$clones,
                                             predClusters = bitTree$clones)


      # Distance (not lo and mb)
      dist[i.rep,"hc"] <- treeDistance(tree1 = dat, tree2 = hcTree,
                                       root2 = which(sapply(hcTree$clones,
                                                            function(x) any(x==0))))
      dist[i.rep,"kc"] <- treeDistance(tree1 = dat, tree2 = kcTree,
                                       root2 = which(sapply(kcTree$clones,
                                                            function(x) any(x==0))))
      dist[i.rep,"lo"] <- NA
      dist[i.rep,"mb"] <- NA
      dist[i.rep,"oncoNEM.est"] <- treeDistance(tree1 = dat,
                                                tree2=oncoTree.est)
      dist[i.rep,"oncoNEM"] <- treeDistance(tree1 = dat,
                                            tree2=oncoTree)
      dist[i.rep,"bitPhylogeny"] <- treeDistance(tree1 = dat,
                                                 tree2 = bitTree)

    }

    RES$vMES[[i.vary]] <- vMes
    RES$DIS[[i.vary]] <- dist
  }

  ## save results
  save(RES,file=paste0('Res/v_',varyX,'.RData'))
}
```

Finally, we apply Kim and Simon's method to the simulated data set and compare its performance with that if oncoNEM. Their method infers onocogenetic trees and does not provide the position of the single cells within the tree. Therefore, V-measure and pairwise cell shortest-path distance cannot be aplied here. Instead, we calculate the average of

a) the fraction of correctly inferred pairwise mutation orders (i.e. mutation a is upstream of mutation b or vice versa) and

b) the fraction of correctly inferred mutually exclusive mutations (i.e. mutations a and b lie on separate branches)

out of all mutations that are not in the same mutation cluster in the simulated tree. This restricition is necessary because Kim and Simon's method does not cluster mutations.

```
if (!file.exists('Res/res_KimAndSimon.RData')) {

  res <- as.data.frame(matrix(0,ncol=6,nrow=sum(sapply(vary,
                                                       function(x) length(get(paste0('v.',x))))*n.rep)),
                       stringsAsFactors = FALSE)
  colnames(res) <- c('varyX',
                     'v.X',
                     'i.rep',
                     'subsetPerformance',
```

```
                    'mutExclusivePerformance',
                    'overallPerformance')

counter=0

for (varyX in vary) {
  v.X <- get(paste0('v.',varyX))

  for (i.vary in 1:length(v.X)) {

    for (i.rep in 1:n.rep) {
      counter <- counter + 1

      load(paste0('SimData/v_',varyX,'-',v.X[i.vary],'_rep',i.rep,'.RData'))

      ## Run Kim and Simon's method
      kimdir <- paste0('Res/Kim/MethodComparison/v_',varyX,'-',v.X[i.vary],'_rep',i.rep)

      dir.create(kimdir,recursive = TRUE,showWarnings = FALSE)

      ## order data by cell index
      mc <- dat$D[,order(as.numeric(colnames(dat$D)))]
      Gob <- inferKimAndSimon(mc=mc, fdr= dat$FPR, ado =  dat$FNR, fileNameBase = kimdir)

      ## Load OncoNEM results
      load(paste0('Res/MethodComparison/oncoNEM_result_v_',varyX,'-',v.X[i.vary],
                  '_rep',i.rep,'.RData'))

      ## Calculate transitive closure of Kim & Simon's inferred mutation graph
      el.Kim <- apply(Gob$edgeList,1,as.numeric)
      tc.Kim <- transitiveClosure(el.Kim)
      ## order by mutation timing from early to late
      tc.Kim <- tc.Kim[order(dat$theta),order(dat$theta)]

      ## Calculate transitive closure of inferred oncoNEM (cell) tree
      el.OncoNEM <- get.edgelist(oncoTree.est$g)
      tc.OncoNEM <- transitiveClosure(el.OncoNEM)
      # extend to mutations
      p_theta.est <- oncoNEMposteriors(tree=oncoTree.est$g, clones=oncoTree.est$clones,
                                       Data=dat$D,FPR=fpr.est,FNR=fnr.est)$p_theta
      theta.est <- apply(p_theta.est,1,which.max)
      tc.OncoNEM <- tc.OncoNEM[theta.est,theta.est]
      ## order by mutation timing
      tc.OncoNEM <- tc.OncoNEM[order(dat$theta),order(dat$theta)]

      ## Calculate transitive closure of simulated cell tree
      el.Sim <- get.edgelist(dat$g)
      tc.Sim <- transitiveClosure(el.Sim)
      # extend to mutations
      tc.Sim <- tc.Sim[dat$theta,dat$theta]
      ## order by mutation timing
      tc.Sim <- tc.Sim[order(dat$theta),order(dat$theta)]

      ## To asses performance we only consider mutations that belong to different clusters in the
      ## simulated trees as Kim & Simon's method does not perform clustering
      mutInDiffClust <- outer(dat$theta,dat$theta, FUN='!=')
```

```
        mutInDiffClust <- mutInDiffClust[order(dat$theta),order(dat$theta)]
        mutInDiffClust[lower.tri(mutInDiffClust)] <- FALSE

        ## Kim and Simon's method
        ## Calculate what fraction of subset relationships is correctly identified
        res$KS.subsetPerformance[counter] <- sum((tc.Sim==tc.Kim)[mutInDiffClust&tc.Sim==1])/
          sum(mutInDiffClust&tc.Sim==1)
        ## Calculate what fraction of mutually exclusive mutations is correctly identified
        res$KS.mutExclusivePerformance[counter] <- sum((tc.Sim==tc.Kim)[mutInDiffClust&tc.Sim!=1])/
          sum(mutInDiffClust&tc.Sim!=1)

        ## oncoNEM
        ## Calculate what fraction of subset relationships is correctly identified
        res$ON.subsetPerformance[counter] <- sum((tc.Sim==tc.OncoNEM)[mutInDiffClust&tc.Sim==1])/
          sum(mutInDiffClust&tc.Sim==1)
        ## Calculate what fraction of mutually exclusive mutations is correctly identified
        res$ON.mutExclusivePerformance[counter] <- sum((tc.Sim==tc.OncoNEM)[mutInDiffClust&tc.Sim!=1])/
          sum(mutInDiffClust&tc.Sim!=1)

        res[counter,c('varyX','v.X','i.rep')] <- c(varyX,v.X[i.vary],i.rep)

      }
      ## Average those 2 performance measures
      res$KS.overallPerformance <- 1/2*(res$KS.subsetPerformance+res$KS.mutExclusivePerformance)
      res$ON.overallPerformance <- 1/2*(res$ON.subsetPerformance+res$ON.mutExclusivePerformance)

    }
  }

  save(res,file = 'Res/res_KimAndSimon.RData')
}
```

3.1 Figure 3B

For every simulation run we extract the error rates that were estimated by oncoNEM and store them in a data frame with all other simulation parameters.

```
estimatedParams <- data.frame(varyX=character(),
                              v.X=numeric(),
                              rep=numeric(),
                              trueFPR=numeric(),
                              estFPR=numeric(),
                              trueFNR=numeric(),
                              estFNR=numeric(),
                              stringsAsFactors = FALSE)


for (varyX in vary) {
  v.X <- get(paste0('v.',varyX))

  for (i.vary in 1:length(v.X)) {

    for (i.rep in 1:n.rep) {
      ## load estimated oncoNEM
      load(paste0('Res/MethodComparison/oncoNEM_result_v_',varyX,'-',
                  v.X[i.vary],'_rep',i.rep,'.RData'))
      if (varyX=='FPR') {
        estimatedParams[nrow(estimatedParams)+1,] <-
          data.frame(varyX=varyX,
                     v.X=v.X[i.vary],
                     rep=i.rep,
                     trueFPR=v.FPR[i.vary],
                     estFPR=fpr.est,
                     trueFNR=fnr,
                     estFNR=fnr.est,stringsAsFactors = FALSE)
      } else if (varyX=='FNR') {
        estimatedParams[nrow(estimatedParams)+1,] <-
          data.frame(varyX=varyX,
                     v.X=v.X[i.vary],
                     rep=i.rep,
                     trueFPR=fpr,
                     estFPR=fpr.est,
                     trueFNR=v.FNR[i.vary],
                     estFNR=fnr.est,
                     stringsAsFactors = FALSE)
      } else {
        estimatedParams[nrow(estimatedParams)+1,] <-
          data.frame(varyX=varyX,
                     v.X=v.X[i.vary],
                     rep=i.rep,
                     trueFPR=fpr,
                     estFPR=fpr.est,
                     trueFNR=fnr,
                     estFNR=fnr.est,
                     stringsAsFactors = FALSE)
      }
    }
  }
}
```

Then we plot the results using `ggplot2`.

```
## prepare data frame for ggplot2
df <- melt(estimatedParams,id.vars =c('varyX','v.X','rep','trueFPR','trueFNR'),
           measure.vars = c('estFPR','estFNR'))
## make single column for ground truth parameter that contains true FPR if the
## measured variable is estFPR and that contains true FNR if the measured
## variable is est FNR>
df$trueValue <- df$trueFPR
df$trueValue[df$variable=='estFNR'] <- df$trueFNR[df$variable=='estFNR']
## add column for faceting
df$variedParam <- paste0(df$varyX,'=',df$v.X)
## set order of facets
df$variedParam <- factor(df$variedParam,levels=unique(df$variedParam))
# if (short) {
#   df£variedParam <- factor(df£variedParam,levels=
#                              paste0(vary,"=",
#                                     unlist(lapply(paste0('v.',vary),get))))
# } else {
#   df£variedParam <- factor(df£variedParam,levels=
#                              paste0(rep(vary,each=4),"=",
#                                     unlist(lapply(paste0('v.',vary),get))))
# }

## set colour of dots to same as for this method in the method comparison plot
df$col <- factor(x=2,levels=1:5)

## make ggplot
ggplot(df,aes(x=rep,y=value,color=col)) +
    stat_summary(mapping=aes(x=3,y=trueValue),
               fun.y=mean,
               fun.ymin=mean,
               fun.ymax=mean,
               geom="crossbar",
               width = 5,
               fatten = 1,
               size=1,
               colour=gg_color_hue(6)[4]) +
  geom_point(alpha=1) +
  facet_grid(variable~variedParam,scales="free_y") +
  scale_colour_manual(values=gg_color_hue(7)[2:3]) +
  scale_y_continuous( breaks=test.fpr,
                      minor_breaks=NULL,
                      limits=c(0,0.5)) +
  scale_x_continuous(limits=c(0.5,n.rep+0.5)) +
  theme_bw()+
  theme(axis.ticks.x = element_blank(),
        axis.text.x = element_blank(),
        axis.title.x = element_blank(),
        axis.title.y = element_blank(),
        panel.grid.minor.x=element_blank(),
        panel.grid.major.x=element_blank(),
        panel.grid.major = element_line(size = 1),
        strip.text.x = element_text(size=10),
        legend.position="none")
```

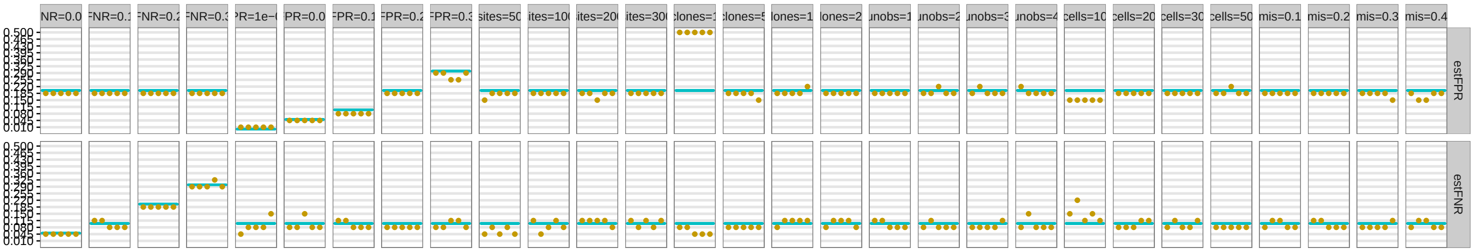

The results show that oncoNEM estimates model parameters accurately over a wide range of simulation settings.

3.2 Figure 5

Next we summarize the performance results of all methods but Kim and Simon's in a single data frame which we then use for plotting. Note that, in order to make the range of values more similar between the different simulated data sets, we normalize the distances for the number of cells in the trees.

```
## initialize df to store results in plotting format
summary <- data.frame(variedParam=factor(),
                      Method=character(),
                      variable=character(),
                      value=numeric())

for (varyX in vary) {

  ## read data for every varied parameter
  load(paste0('Res/v_',varyX,'.RData'))
  ## change data structure
  dist <- as.data.frame(cbind(RES$DIS[[1]],variedParam=get(paste0('v.',varyX))[1]))
  vMes <- as.data.frame(cbind(RES$vMES[[1]],variedParam=get(paste0('v.',varyX))[1]))
  if (!short){
    for (i in 2:length(RES$DIS)) {
      dist <- rbind(dist,cbind(RES$DIS[[i]],variedParam=get(paste0('v.',varyX))[i]))
      vMes <- rbind(vMes,cbind(RES$vMES[[i]],variedParam=get(paste0('v.',varyX))[i]))
    }
  }
  ## adjust distance for number of cells
  if (varyX!="cells") {
    dist[,1:(ncol(dist)-1)] <- dist[,1:(ncol(dist)-1)]/(((n.cells+2)*(n.cells+1))/2)
        ## +1 for cell added to root by treeDistance
        ## +1 for normal cell added to each simulated data set
  } else {
    dist[,1:(ncol(dist)-1)] <- dist[,1:(ncol(dist)-1)]/(((dist$variedParam+2)*(dist$variedParam+1))/2)
  }
  ## melt
  dist_melt <- melt(dist,id="variedParam",variable.name = "Method")
  vMes_melt <- melt(vMes,id="variedParam",variable.name = "Method")
  ## combine distance and v-measure
  res <- cbind(dist_melt,vMes_melt$value)
  colnames(res)[3:4] <- c("Distance/(N*(N-1)/2)","V-measure")
  ## melt again
  res <- melt(res,id=c('variedParam','Method'))
  ## set order of facets in plot
  res$variedParam <- factor(paste0(varyX,'=',res$variedParam),
                            levels=unique(paste0(varyX,'=',res$variedParam)))
  summary <- rbind(summary,res)

}
```

Now we plot the results of the method comparison obtained above.

```
## change levels
summary$Method <- factor(summary$Method,levels=c("oncoNEM","oncoNEM.est",
                                                 "bitPhylogeny","hc","kc","lo","mb"))

## remove missing values for failed inferences
summary <- summary[!is.na(summary$value),]
```

```r
ggplot(summary[summary$variable=='Distance/(N*(N-1)/2)',], aes(x=Method,y=value))+
  geom_dotplot(aes(y = value, x = Method, fill = Method , colour = Method),
               binaxis = "y",
               binwidth = 0.05,
               stackdir = "center",
               dotsize = 3,
               stackratio = 0.5) +
  stat_summary(fun.y=mean,
               fun.ymin=mean,
               fun.ymax=mean,
               geom="crossbar",
               width = 1,
               fatten = 1,
               colour='black') +
  scale_y_reverse() +
  facet_grid(variable~variedParam,scales="free_y")+
  theme_bw() +
  theme(axis.ticks.x = element_blank(),
        axis.text.x = element_blank(),
        axis.title.x = element_blank(),
        axis.title.y = element_blank(),
        panel.grid.minor.x=element_blank(),
        panel.grid.major.x=element_blank(),
        legend.position="bottom") +
  guides(fill=FALSE)

ggplot(summary[summary$variable=='V-measure',], aes(x=Method,y=value))+
  geom_dotplot(aes(y = value, x = Method, fill = Method, colour = Method),
               binaxis = "y",
               binwidth = 0.05,
               stackdir = "center",
               dotsize = 1,
               stackratio = 0.5) +
  stat_summary(fun.y=mean,
               fun.ymin=mean,
               fun.ymax=mean,
               geom="crossbar",
               width = 1,
               fatten = 1,
               colour='black') +
  facet_grid(variable~variedParam) +
  scale_colour_manual(values=gg_color_hue(7)) +
  scale_y_continuous(limits = c(0, 1)) +
  theme_bw()+
  theme(axis.ticks.x = element_blank(),
        axis.text.x = element_blank(),
        axis.title.x = element_blank(),
        axis.title.y = element_blank(),
        panel.grid.minor.x=element_blank(),
        panel.grid.major.x=element_blank(),
        legend.position="bottom")+
  guides(fill=FALSE)
```

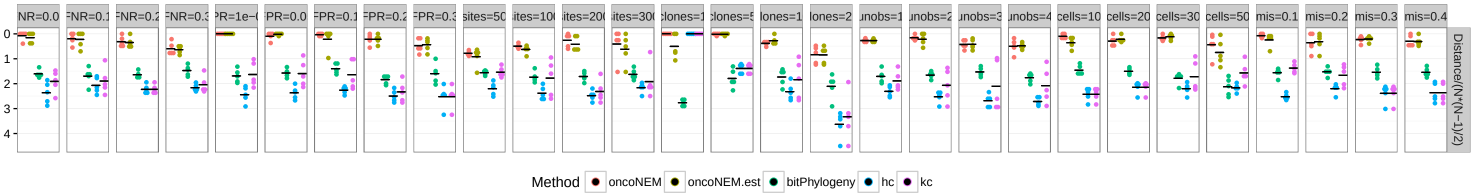

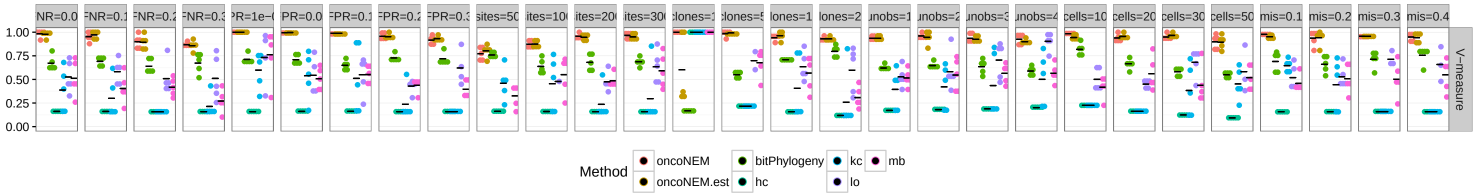

For the comparison with Kim and Simon's method we plot the accuracy of the inferred mutation orders obtained earlier in this section.

```
load('Res/res_KimAndSimon.RData')
res2 <- melt(res,
             id.vars = c('varyX','v.X','i.rep'),
             measure.vars = c('KS.overallPerformance','ON.overallPerformance'))
res2$variedParam <- paste0(res2$varyX,res2$v.X)
res2$variedParam <- factor(res2$variedParam,levels = unique(res2$variedParam))

ggplot(res2,aes(x=i.rep,y=value,col=variable)) +
  facet_grid(.~variedParam,scales="free_x") +
  geom_point(na.rm=TRUE) +
  scale_y_continuous(limits=c(0,1)) +
  theme_bw() +
  theme(axis.ticks.x = element_blank(),
        axis.text.x = element_blank(),
        axis.title.x = element_blank(),
        panel.grid.minor.x = element_blank(),
        panel.grid.major.x = element_blank(),
        panel.grid.major.y = element_line(size = 0.3),
        legend.position='bottom') +
  ylab("Accuracy of mutation order")
```

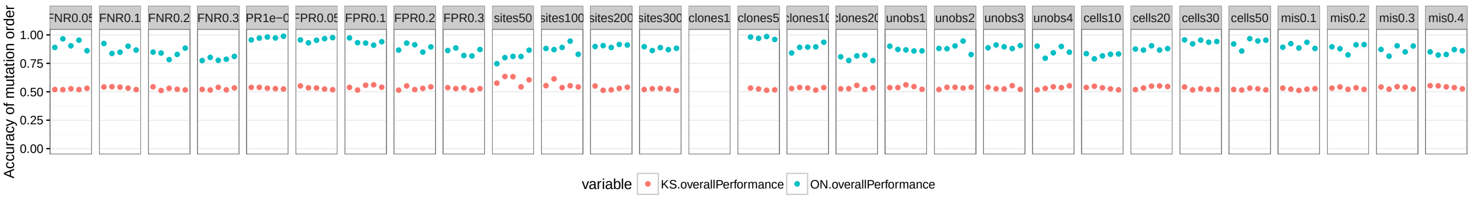

The two plots show that oncoNEM outperforms the baseline methods for all simulation scenarios but the single clone case. It consistently yields results that have a smaller distance to the ground truth and a higher V-measure and the inferred ordering of mutation is more accurate.

4 Dependence of oncoNEM results on threshold ϵ (Figure 4)

For both the simulation studies and the case studies, the Bayes' factor threshold ϵ, which is used by oncoNEM during the clustering, was set to 10. To evaluate the effect of this parameter on the oncoNEM results, we repeat the oncoNEM inference on all data sets from the method comparison studies while varying ϵ from 1 to 20. For each simulated data set and ϵ we calculate the V-measure, the normalized distance and the number of clones of the inferred solution.

```
epsilon <- c(1,2,5,10,15,20)
if (!file.exists('Res/resEpsilon.RData')) {
  counter <- 0
  resEpsilon <- data.frame(epsilon = rep(epsilon,
                                         sum(sapply(vary,function(x) length(get(paste0('v.',x))))*n.rep)),
                          varyX='X', vX = 0,vMeasure = 0, dist = 0, stringsAsFactors=FALSE,nClones=0)

  for (varyX in vary) {
    v.X <- get(paste0('v.',varyX))

    for (i.vary in 1:length(v.X)) {

      for (i.rep in 1:n.rep) {

        ## load data
        load(paste0('SimData/v_',varyX,'-',v.X[i.vary],'_rep',i.rep,'.RData'))

        ## load res
        load(paste0('Res/MethodComparison/oncoNEM_result_v_',varyX,
                    '-',v.X[i.vary],'_rep',i.rep,'.RData'))

        ## infer unclustered tree
        ## ----------------------------------------------------------------------
        ## Run oncoNEM with estimated Parameters
        ## initialize oncoNEM
        oNEM.est <- oncoNEM$new(Data=dat$D,
                                FPR=fpr.est,
                                FNR=fnr.est)
        ## Run search algorithm until best tree has not changed for at least
        ## delta steps.
        oNEM.est$search(delta=200)

        for (e in epsilon) {
          counter <- counter + 1

          # search for hidden nodes
          oNEM.est.expanded <- expandOncoNEM(oNEM.est,epsilon = e,delta = 200)

          # cluster
          oncoTree.est <- clusterOncoNEM(oNEM.est.expanded, epsilon=e)

          # relabel cells from column indices to cell indices as in ground truth
          # tree
          oncoTree.est$clones <- relabelCells(clones = oncoTree.est$clones,
                                              labels = as.numeric(colnames(dat$D)))

          ## evaluate performance
          resEpsilon$epsilon[counter] <- e
          resEpsilon$varyX[counter] <- varyX
          resEpsilon$vX[counter] <- v.X[i.vary]
```

```
          resEpsilon$vMeasure[counter] <- vMeasure(trueClusters = dat$clones,
                                                   predClusters = oncoTree.est$clones)
          resEpsilon$dist[counter] <- treeDistance(tree1 = dat,
                                                   tree2=oncoTree.est)
          resEpsilon$nClones[counter] <- length(oncoTree.est$clones)
        }
        rm(oNEM.est)
        rm(oNEM.est.expanded)
        save(resEpsilon,file='Res/resEpsilon.RData')
      }
    }
  }

}
```

Finally we plot these three measures as a function of epsilon for the different simulation scenarios.

```
load('Res/resEpsilon.RData')

## assign id for each line
resEpsilon$id <- rep(1:(nrow(resEpsilon)/length(epsilon)),each=length(epsilon))
## assign id for colouring each line by parameter setting
resEpsilon$id2 <- as.factor(unlist(lapply(vary,function(x) rep(1:length(get(paste0('v.',x))),
                                                               each=length(epsilon)*n.rep))))

## normalize distance by number of cell
resEpsilon$dist[resEpsilon$varyX!='cells'] <- resEpsilon$dist[resEpsilon$varyX!='cells']/
  (((n.cells+2)*(n.cells+1))/2)
  ## +1 for cell added to root by treeDistance
  ## +1 for normal cell added to each simulated data set
nv.cells <- resEpsilon$vX[resEpsilon$varyX=='cells']
resEpsilon$dist[resEpsilon$varyX=='cells'] <- resEpsilon$dist[resEpsilon$varyX=='cells']/
  (((nv.cells+2)*(nv.cells+1))/2)


resEpsilon <- melt(resEpsilon,id.vars = c('epsilon','varyX','vX','id','id2'),
                   measure.vars = c('vMeasure','nClones'))
levels(resEpsilon$variable) <- c('V-measure','# clones')

ggplot(resEpsilon,aes(x=epsilon,y=value,colour=id2)) +
  geom_line(aes(group=id)) +
  facet_grid(variable~varyX,scales="free_y")  +
  scale_color_discrete(name="Parameter\nSetting") +
  expand_limits(y=0) +
  geom_vline(xintercept=10,linetype=2) +
  theme_bw() +
  theme(axis.title.y = element_blank(),legend.position='bottom')
```

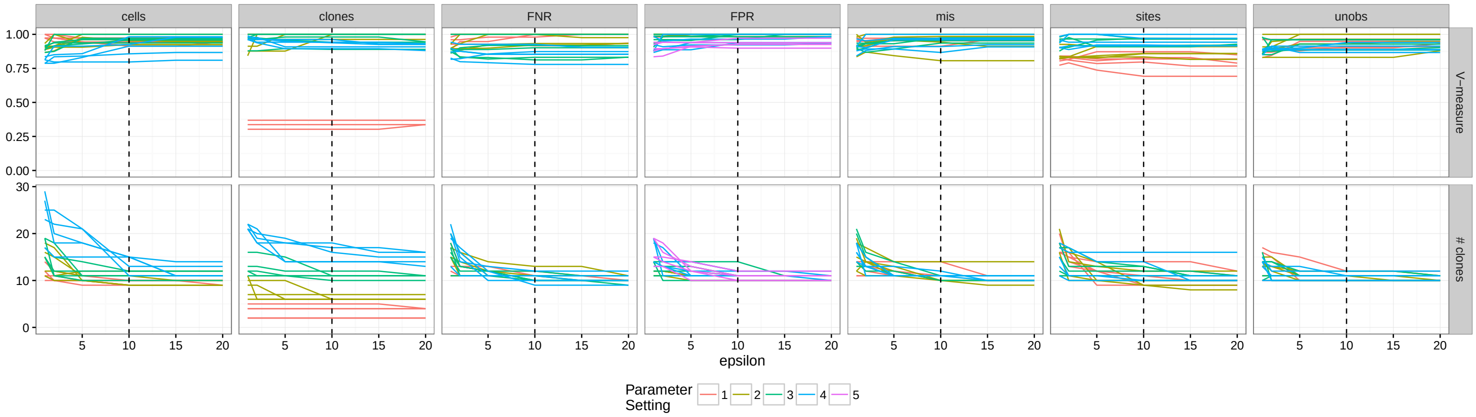

In all simulation scenarios the number of clones is largely independent of epsilon, unless for unreasonably small choices of $\epsilon < 5$. The threshold for ϵ used throughout the paper is 10 (dashed line), and thus well within the stable range.

5 Session Info

```r
sessionInfo()

## R version 3.2.3 (2015-12-10)
## Platform: x86_64-pc-linux-gnu (64-bit)
## Running under: Ubuntu 14.04.3 LTS
##
## locale:
##  [1] LC_CTYPE=en_GB.UTF-8       LC_NUMERIC=C
##  [3] LC_TIME=en_GB.UTF-8        LC_COLLATE=en_GB.UTF-8
##  [5] LC_MONETARY=en_GB.UTF-8    LC_MESSAGES=en_GB.UTF-8
##  [7] LC_PAPER=en_GB.UTF-8       LC_NAME=C
##  [9] LC_ADDRESS=C               LC_TELEPHONE=C
## [11] LC_MEASUREMENT=en_GB.UTF-8 LC_IDENTIFICATION=C
##
## attached base packages:
## [1] parallel  stats     graphics  grDevices utils     datasets  methods
## [8] base
##
## other attached packages:
##  [1] KimAndSimon_0.0.0.9000 RBGL_1.44.0            graph_1.48.0
##  [4] phangorn_1.99.14       ape_3.3                cluster_2.0.3
##  [7] doParallel_1.0.10      iterators_1.0.8        foreach_1.4.3
## [10] bitphylogenyR_0.99     igraph_1.0.1           rPython_0.0-5
## [13] RJSONIO_1.3-0          reshape2_1.4.1         ggplot2_2.0.0
## [16] oncoNEM_1.0            Rcpp_0.12.3            knitr_1.10.5
##
## loaded via a namespace (and not attached):
##  [1] formatR_1.2        plyr_1.8.3         highr_0.5
##  [4] bitops_1.0-6       class_7.3-14       tools_3.2.3
##  [7] digest_0.6.9       evaluate_0.7       gtable_0.2.0
## [10] nlme_3.1-123       lattice_0.20-33    Matrix_1.2-3
## [13] e1071_1.6-7        stringr_1.0.0      gtools_3.5.0
## [16] caTools_1.17.1     stats4_3.2.3       grid_3.2.3
## [19] gdata_2.17.0       magrittr_1.5       BiocGenerics_0.16.1
## [22] scales_0.4.0       gplots_2.17.0      codetools_0.2-14
## [25] nnls_1.4           riverplot_0.5      mcclust_1.0
## [28] colorspace_1.2-6   labeling_0.3       quadprog_1.5-5
## [31] KernSmooth_2.23-15 stringi_1.0-1      munsell_0.4.3
## [34] ggm_2.3
```
