# Supplementary material for: OncoNEM: inferring tumor evolution from single-cell sequencing data
Source: Genome Biol. 2016 Apr 15;17:69. doi: 10.1186/s13059-016-0929-9 (PMC4832472; doi:10.1186/s13059-016-0929-9)
Supplement: Additional file 5 — PDF of compiled knitr script for case studies. A PDF file reproducing the results of the case studies. (PDF 463 kb) [file 13059_2016_929_MOESM5_ESM.pdf]

Supplement to
OncoNEM: Inferring tumour evolution from single-cell sequencing data
# – Case studies –

Edith M Ross and Florian Markowetz

March 10, 2016

1 Settings

To rerun the complete analysis of the case studies set the variable `short` in the following code chunk to `FALSE`. If it is set to `TRUE` the parameter estimation step is skipped and fixed parameter settings are used.

```
short <- FALSE
```

2 Data overview and pre-processing

First, we load the R packages used in this vignette.

```
options(stringsAsFactors = FALSE,scipen = 8)
library(oncoNEM)

## Loading required package:  Rcpp

library(ggplot2)
library(xtable)
```

This automatically loads the data that is included in the oncoNEM package. The data files are pre-processed versions of the data provided in the paper supplements by Li et al [?] and Hou et al [?].

Alternatively, to generate the pre-processed data from the original files, download additional file 5 from Li et al [?] and supplementary table S4 from Hou et al [?], save them in a directory named `'./Data'` and run the following two code chunks.

These chunks binarize the data, select all columns that correspond to single tumour cells and read in additional annotation data.

```
## load and reformat data
library(gdata)
if (!file.exists('Data/2047-217x-1-12-s5.xls')) {
  stop("Additional file 5 from Li et al (2012) not found.")
}

D <- gdata::read.xls('Data/2047-217x-1-12-s5.xls',sheet=3,skip=3)
Li <- D[,-1]
rownames(Li) <- D[,1]
colnames(Li) <- gsub('\\.','-',colnames(Li))

## set missing values
Li[Li=="-"] <- NA

## indices of normal cells
```

```r
indx.normal <- grep('BN',colnames(Li))

normal.gtyp <- apply(Li[,indx.normal],1,function(x) unique(x[!is.na(x)]))

## select only single cancer cells
Li <- Li[,-c(1,indx.normal)]

## convert to binary matrix
D <- t(sapply(1:nrow(Li), function(i) Li[i,]!=normal.gtyp[i])*1)
rownames(D) <- rownames(Li)
colnames(D) <- colnames(Li)

## set missing entries to 2
D[is.na(D)] <- 2
## save as Li
Li <- D

## load data for recurrently mutated genes
annot <- gdata::read.xls('Data/2047-217x-1-12-s5.xls',sheet=2,skip=3)
colnames(annot)[1] <- 'coord'
geneName <- c('NIPBL','CFTR','DHX57','ASTN1','COL6A3','KIAA1958','ATM')
recurrGenes <- annot[annot$Gene.Name%in%geneName,]
Li.recurrGenes <- cbind(recurrGenes,gtyp.indx=match(recurrGenes$coord,rownames(D)))

## extract annotation data (cell IDs and matched clone ID) from Figure 2A
Li.annot <- data.frame(BCid=colnames(Li),cloneid=NA)
Li.annot$cloneid[Li.annot$BCid%in%c('BC-52','BC-33','BC-24','BC-47','BC-16',
                                    'BC-8','BC-37','BC-9','BC-11','BC-43',
                                    'BC-7','BC-42','BC-50','BC-58','BC-59')] <- 'A'
Li.annot$cloneid[Li.annot$BCid%in%c('BC-56','BC-35','BC-55','BC-36','BC-15',
                                    'BC-53','BC-48','BC-22','BC-32','BC-49',
                                    'BC-45','BC-25','BC-51')] <- 'B'
Li.annot$cloneid[Li.annot$BCid%in%c('BC-21','BC-46','BC-34','BC-38','BC-18',
                                    'BC-6','BC-31','BC-28','BC-39','BC-13',
                                    'BC-41','BC-54','BC-29','BC-44','BC-14',
                                    'BC-23')] <- 'C'
```

```r
## load and reformat data

if (!file.exists('Data/mmc2.xls')) {
  stop("Supplementary table S4 from Hou et al (2012) not found.")
}

D <- read.xls('Data/mmc2.xls',sheet=1,skip=2)
## remove last two lines (contain comments)
D <- D[-(c(-1,0)+nrow(D)),]
## remove first column (contains coordinates) and assign as rownames
Hou <- D[,-1]
rownames(Hou) <- D[,1]
## make column names consistent
colnames(Hou) <- gsub('\\.','-',colnames(Hou))

## set missing sites to NA
Hou[Hou=="-"] <- NA
```

```r
## index of normal tissue
indx.normal <- grep('LN',colnames(Hou))

## define normal genotype
normal.gtyp <- Hou[,indx.normal]

## Convert to binary matrix
## Assumption for GTYP -> SNV conversion:
## Under infinite sites model, each position can only be mutated once,
## i.e. two genotypes are possible: homozygous wt/wt (all wt genotypes
## are homozygous in our dataset) or heterozygous wt/mut. If we observe
## a homozygous mut/mut genotype we assume that the true underlying
## genotype is wt/mut.
D <- t(apply(Hou,1,function(x) x!=x[indx.normal])*1)
rownames(D) <- rownames(Hou)

## only select single cancer cells:
D <- D[,grep('LC-[0-9]',colnames(Hou))]
## set missing entries to 2
D[is.na(D)] <- 2

## save with different name
Hou <- D
```

The bladder cancer (TCC) data set contains 44 cells and 443 mutations.

```r
dim(Li)

## [1] 443  44

Li[1:5,1:5]

##                   BC-6 BC-7 BC-8 BC-9 BC-11
## 1,1411704,1,G/C     1    0    0    2     2
## 1,7756132,1,C/T     2    2    1    2     2
## 1,9958226,1,T/C     0    1    0    1     1
## 1,11109449,1,A/G    0    2    2    1     2
## 1,12627344,1,C/T    2    1    1    1     2
```

The essential thrombocythemia (ET) data set contains 58 cells and 712 mutations.

```r
dim(Hou)

## [1] 712  58

Hou[1:5,1:5]

##                   LC-1 LC-100 LC-12 LC-16 LC-18
## 1,109658366,1,G/T    0      2     2     1     2
## 1,11657832,1,A/C     2      1     1     1     2
## 1,116944223,1,C/A    0      0     0     0     2
## 1,116948115,1,C/G    0      0     0     0     2
## 1,11728503,1,T/C     0      2     0     2     0
```

Both data set contains values from 0 to 2, where 0 denotes an non-mutant site, 1 denotes a mutation and 2 denotes a missing value.

```r
table(Li)/prod(dim(Li))

## Li
##         0         1         2
## 0.2746255 0.1731480 0.5522266

table(Hou)/prod(dim(Hou))

## Hou
##         0         1         2
## 0.1799932 0.2427838 0.5772230
```

3 OncoNEM Inference

With the pre-processed data we can run the oncoNEM inference algorithm on both data sets.

3.1 Parameter estimation

The first step is to estimate the model parameters, i.e. FPR and FNR. This is a computationally expensive step. To reduce the compilation time of this document, this step is skipped and previously computed results are used by default. This can be changed by setting `runParamEst` in the next code chunk to `TRUE`.

```r
runParamEst <- TRUE ## Set this to TRUE if the parameter estimation should be
                     ## rerun. Otherwise the previously estimtated values will be
                     ## used.

## Names of the two data sets
ds <- c('Hou','Li')

for (dsX in ds) {
  D <- get(dsX)
  colnames(D) <- 1:ncol(D)

  if (short & dsX=='Hou') {
    test.fpr <- c(0.22,0.255,0.29)
    test.fnr <- c(0.15,0.185,0.22)
  } else if (short & dsX=='Li') {
    test.fpr <- c(0.15,0.185,0.22)
    test.fnr <- c(0.045,0.08,0.115)
  } else {
    test.fpr <- test.fnr <- seq(from=0.01,to=0.5,length.out=15)
  }

  ## ----------------------------------------------------------------------------
  ## Parameter estimation
  if (runParamEst) {
    dir.create(paste0('Res/',dsX),recursive = TRUE,showWarnings = FALSE)

    llh <- matrix(0,nrow = length(test.fpr), ncol=length(test.fnr))
    trees <- array(dim = c(length(test.fpr),length(test.fnr),ncol(D)))
    for (i.fnr in 1:length(test.fnr)) {
      for (i.fpr in 1:length(test.fpr)) {

        ## infer oncoNEM
        oNEM <- oncoNEM$new(Data=D,FPR=test.fpr[i.fpr],FNR=test.fnr[i.fnr])
```

```
        oNEM$search(delta=200)
        print(oNEM$TF$getBestCounter( ))

        llh[i.fpr,i.fnr] <- oNEM$best$llh
        trees[i.fpr,i.fnr,] <- oNEM$best$tree

        ## save output in every round in case something goes wrong
        save(llh,trees,file=paste0('Res/',dsX,'/llh_and_trees.RData'))
      }
    }
  } else {
    load(paste0('Res/',dsX,'/llh_and_trees.RData'))
  }

  ##--------------------------------------------------------------------------------
  ## Evaluate results of parameter estimation

  ## find best parameter combination
  indx <- which(llh==max(llh),arr.ind=TRUE)
  fpr.est <- test.fpr[indx[1]]
  fnr.est <- test.fnr[indx[2]]
  param.est <- data.frame(x=fpr.est,y=fnr.est,value="Parameter estimate")

  save(param.est,fpr.est,fnr.est,test.fpr,test.fnr,indx,
       file=paste0('Res/',dsX,'/paramEst.RData'))
}
```

Next we plot these results in form of heatmaps that describe how the log-likelihoods of the highest scoring trees depend on the error parameters.

```
## --------------------------------------------------------------------------------
## Plot llh
for (dsX in ds) {
  ## Plot likelihood distribution for parameter combinations

  load(paste0('Res/',dsX,'/llh_and_trees.RData'))
  load(paste0('Res/',dsX,'/paramEst.RData'))

  if (dsX=='Li') {
    plotTitle <- 'Bladder cancer'
  } else {
    plotTitle <- 'Essential thrombocythemia'
  }

  ## format data for ggplot
  df <- cbind(expand.grid(test.fpr,test.fnr),
              llh[as.matrix(expand.grid(1:length(test.fpr),
                                         1:length(test.fnr)))])
  colnames(df) <- c('fpr','fnr','llh')
  p <- ggplot(df, aes(fpr,fnr)) +
    geom_raster(aes(fill = llh),hjust = 0.5, vjust = 0.5)+
    geom_point(data=param.est,aes(x=x,y=y,shape=value),
               colour="black",bg="white",size=6) +
    scale_shape_manual(values = c('Parameter estimate'=25)) +
    labs(x = 'False positive rate',
         y = 'False negative rate') +
```

```
    scale_fill_gradientn(colours = rainbow(8)[1:7],
                         name='Log-likelihood',
                         limits=c(max(llh)-1000+5,max(llh)+5)) +
    theme(axis.text  = element_text(size=12),
          axis.title = element_text(size=16),
          axis.title.y = element_text(vjust = 1),
          strip.text.x = element_text(size=16),
          legend.text = element_text(size=16),
          legend.title = element_blank(),
          aspect.ratio = 1,
          plot.title = element_text(face="bold")) +
    ggtitle(plotTitle)

  print(p)
}
```

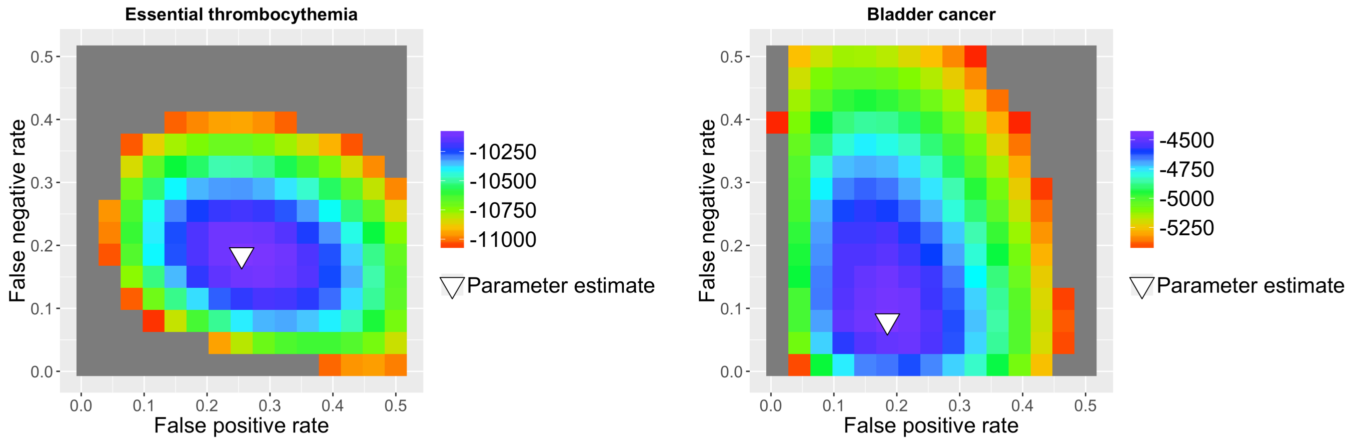

The estimated parameters for the Bladder cancer data are FPR = 0.185 and FNR = 0.08.
For the Essential thrombocythemia data set the estimated error rates are FPR = 0.255 and FNR = 0.185.

3.2 Inferring clonal lineage trees

Next, we infer clonal lineage trees based on the estimated parameters. To be able to assess whether the results are robust, we also infer trees with parameter combinations surrounding our estimate.

```
for (dsX in ds) {
  load(paste0('Res/',dsX,'/llh_and_trees.RData'))
  load(paste0('Res/',dsX,'/paramEst.RData'))

  D <- get(dsX)
  ##-----------------------------------------------------------------------------
  ## Compare tree from estimated parameters to tree from neighboring parameters
  ## to assess stability

  ## choose indices of parameter combinations to test
  fpr.indx.env <- indx[1] + (-1:1)
```

```
  fnr.indx.env <- indx[2] + (-1:1)

  for (i.fpr in fpr.indx.env) {
    for (i.fnr in fnr.indx.env) {

      oNEM <- oncoNEM$new(Data=D,FPR=test.fpr[i.fpr],FNR=test.fnr[i.fnr])
      ## add tree from previous initial search
      oNEM$addTree(trees[i.fpr,i.fnr,])
      ## serach for unobserved nodes
      oNEM.expanded <- expandOncoNEM(oNEM,epsilon = 10,delta = 200,
                                     checkMax = 10000,app=TRUE)
      # cluster
      oncoTree <- clusterOncoNEM(oNEM = oNEM.expanded, epsilon=10)

      ssave(c('oNEM','oNEM.expanded','oncoTree'),
            file=paste0('Res/',dsX,'/inferredTree_fpr-',test.fpr[i.fpr],'_fnr-',
                        test.fnr[i.fnr],'.RData'))
    }
  }

}
```

The two inferred trees are plotted in the following.

```
#<<PlotTrees,out.width='7cm', fig.show='hold'>>=
##-------------------------------------------------------------------------------
## Plot trees

par(mfrow=c(1,2))
for (dsX in ds) {
  load(paste0('Res/',dsX,'/paramEst.RData'))
  load(paste0('Res/',dsX,'/inferredTree_fpr-',fpr.est,'_fnr-',fnr.est,'.RData'))

  ## calculate posterior probabilies of theta for edge length estimation
  post <- oncoNEMposteriors(tree = oncoTree$g,
                            clones = oncoTree$clones,
                            Data = oNEM$Data,
                            FPR = oNEM$FPR,FNR = oNEM$FNR)

  ## set vertex size proportional to size of subpopulations
  vSize <- sapply(oncoTree$clones,length)
  vSize <- vSize/sum(vSize)*200
  vSize[1] <- 20 ## node size of normal

  par(mar=c(1,4,4,2))
  plotTree(tree = oncoTree$g,clones = NULL,e.length = colSums(post$p_theta)[-1],
           label.length = "max",axis=TRUE,vertex.size=vSize,edge.arrow.mode='-',
           vertex.label=NA,vertex.color=c('paleturquoise3',
                                          rep('thistle',igraph::vcount(oncoTree$g)-1)),
           ylab='Accumulated mutations')

  if (dsX=='Li') {
    title(main='Bladder cancer')
  } else {
    title(main='Essential thrombocythemia')
  }
```

```
}
```

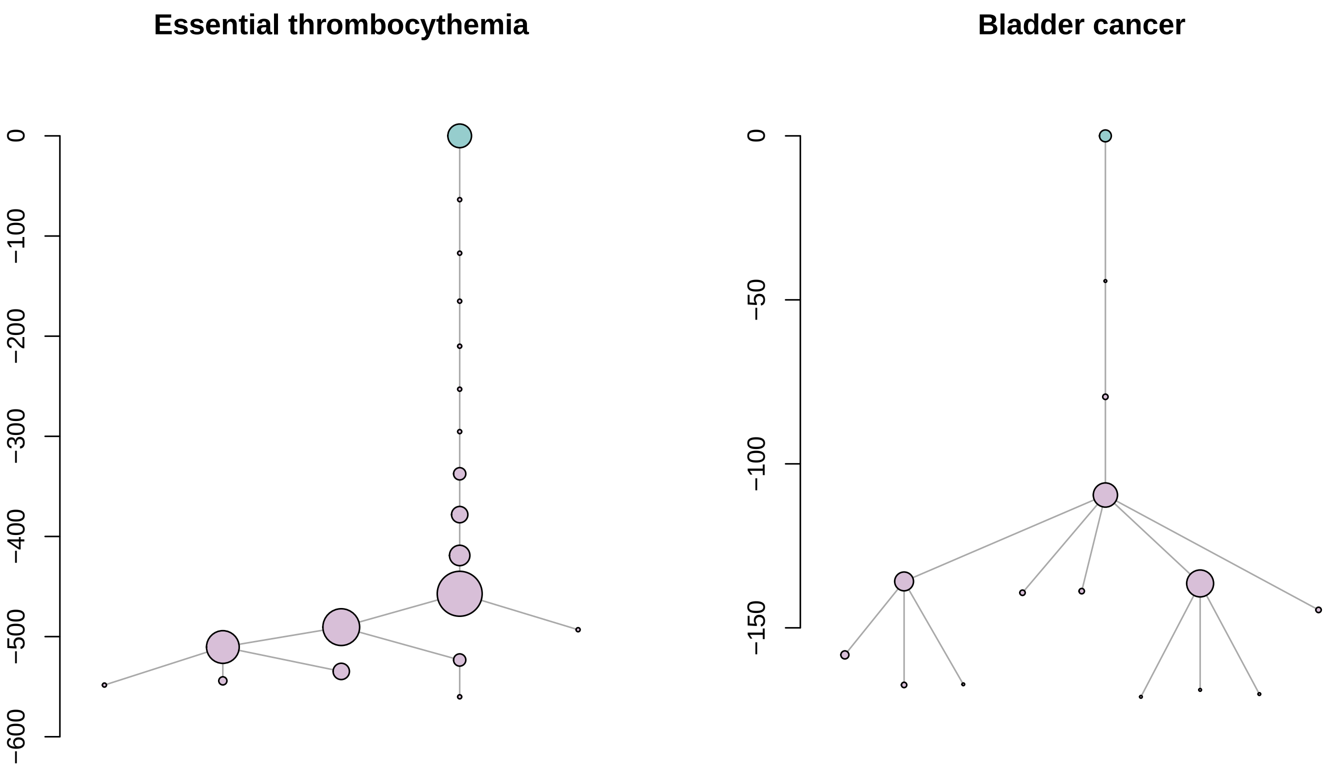

For comparison, we next plot the inferred tree of each data set together with the trees that were inferred using similar parameters.

```
##----------------------------------------------------------------------------
## Plot trees
for (dsX in ds) {
  load(paste0('Res/',dsX,'/paramEst.RData'))

  ## choose indices of parameter combinations
  fpr.indx.env <- indx[1] + (-1:1)
  fnr.indx.env <- indx[2] + (-1:1)

  par(mfrow=c(3,3),mar=c(0.1,2,2,0.1),oma=c(0,0,2,0))

  for (i.fpr in fpr.indx.env) {
    for (i.fnr in fnr.indx.env) {
      load(paste0('Res/',dsX,'/inferredTree_fpr-',test.fpr[i.fpr],'_fnr-',
                  test.fnr[i.fnr],'.RData'))
      Data <- oNEM$Data
      colnames(Data) <- 1:ncol(Data)
      post <- oncoNEMposteriors(tree = oncoTree$g,
                                clones = oncoTree$clones,
                                Data = Data,
                                FPR = oNEM$FPR,FNR = oNEM$FNR)

      plotTree(tree = oncoTree$g,clones = oncoTree$clones,
               e.length = colSums(post$p_theta)[-1],
               label.length = 20,axis=FALSE,edge.arrow.mode='-',
```

```
                vertex.color='white',
                v.label.cex=1.15)
      ## add customized y axis
      if (dsX=='Li') {
        axis(2, labels = seq(0,200,by=50), at = seq(0,-200,by=-50))
      } else {
        axis(2, labels = seq(0,600,by=100), at = seq(0,-600,by=-100))
      }
      title(paste('FPR =', test.fpr[i.fpr],', FNR =',test.fnr[i.fnr],
                  ', LLH =',round(oncoTree$llh,digits = 1)), line = -1.5)
    }
  }
  ## add overall title
  if (dsX=='Li') {
    title(main=list("Bladder cancer",cex=1.5),outer=TRUE)
  } else {
    title(main=list("Essential thrombocythemia",cex=1.5),outer=TRUE)
  }
}
```

Essential thrombocythemia

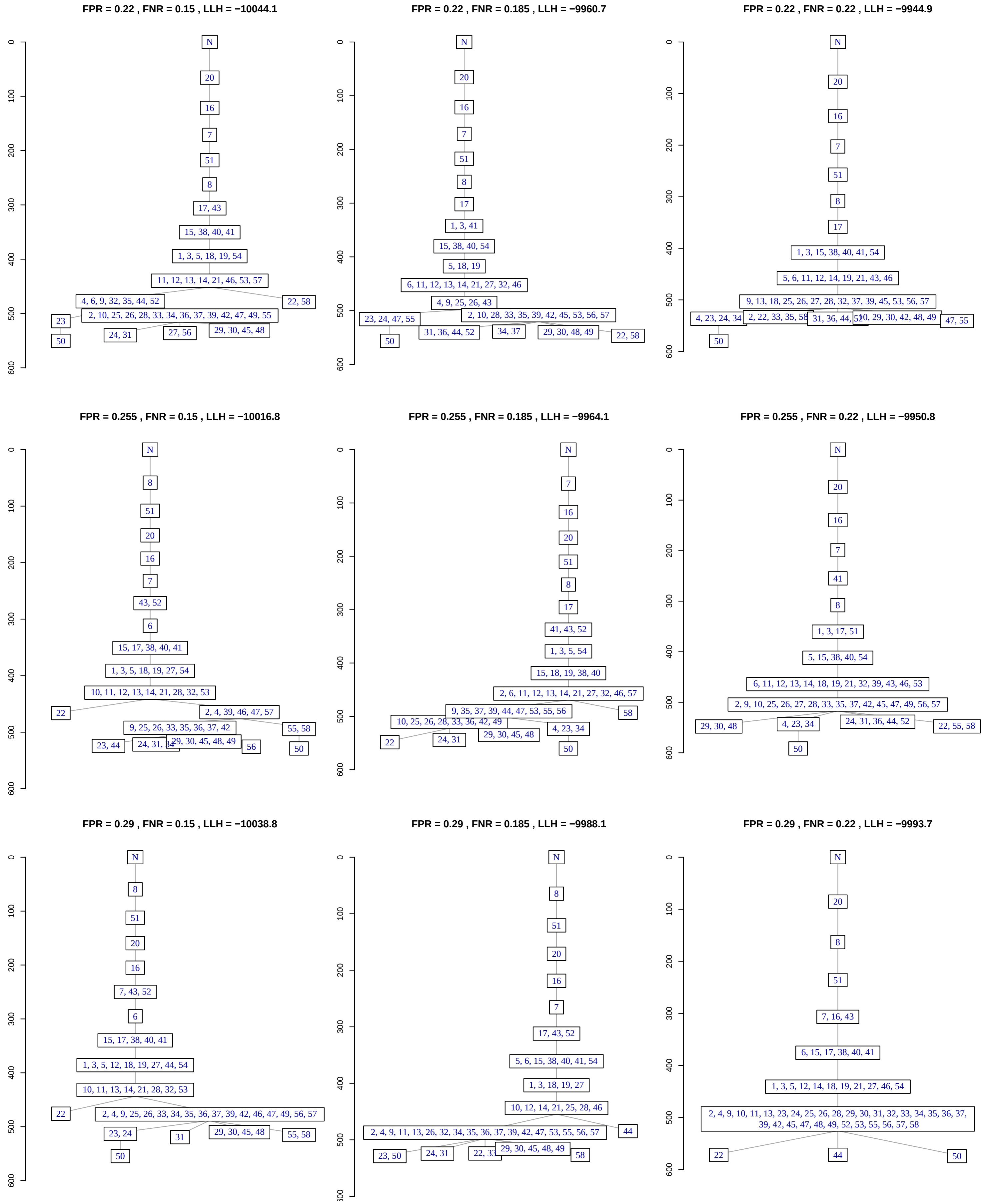

Bladder cancer

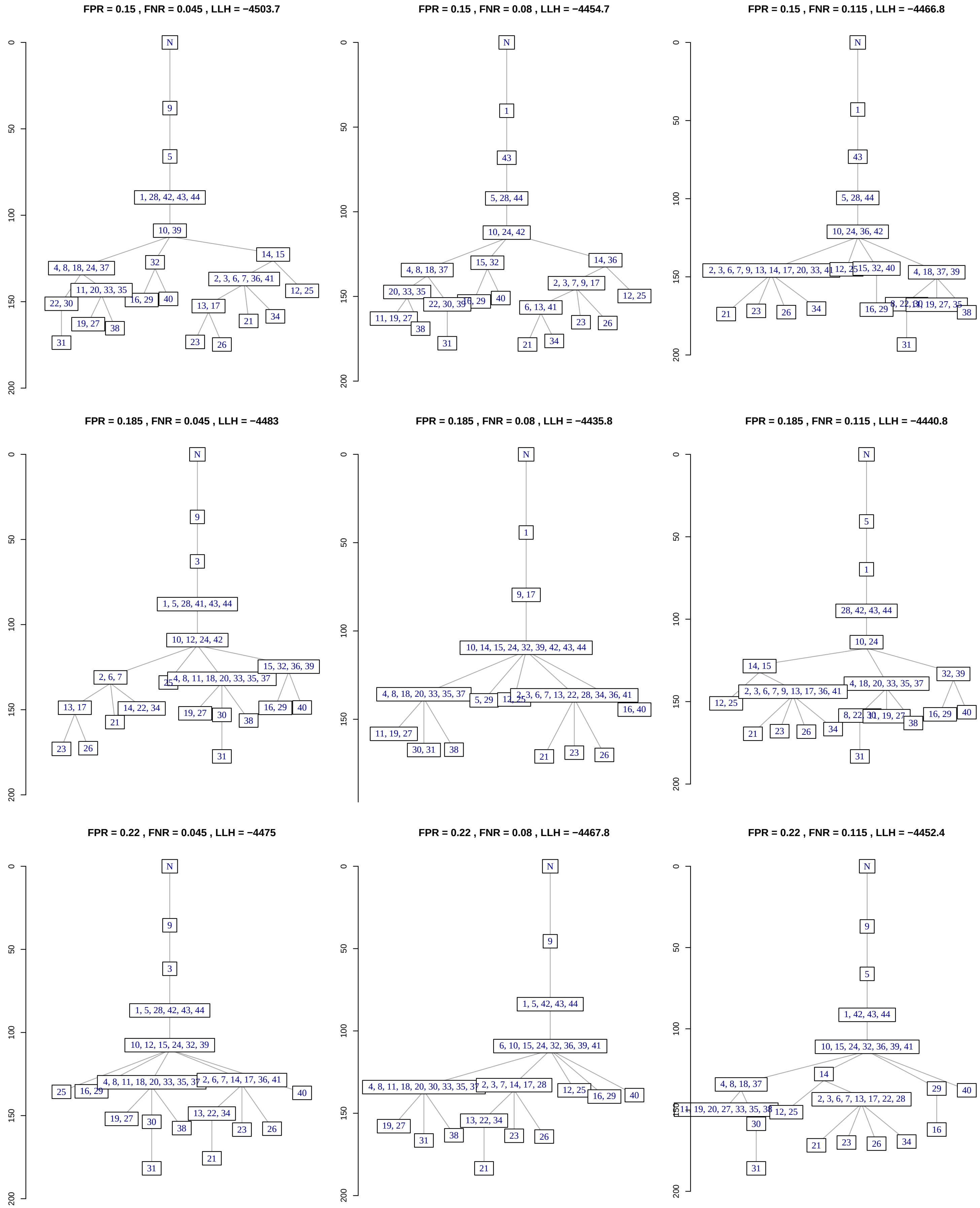

4 TCC — Inference on LOH-blacklisted data set

We repeat the previous analysis on the TCC data set excluding all mutations that lie within large regions of LOH. To do this we first filter the input data set.

```
coords <- rownames(Li)
coords.df <- data.frame(chr=as.numeric(sub(",.*","",coords)),
                        pos=as.numeric(sub(".*,(.*),.*,.*","\\1",coords)))
coords.df$coord <- sub(",.*","",coords)

## exclude
exclude.idx <- c(which(coords.df$chr==2&coords.df$pos>205600000),
                 which(coords.df$chr==9),
                 which(coords.df$chr==10&coords.df$pos>114900000),
                 which(coords.df$chr==11&coords.df$pos<52900000),
                 which(coords.df$chr==22&coords.df$pos>11800000))

## remove mutations in LOH regions:
D <- Li[-exclude.idx,]
colnames(D) <- 1:ncol(D)
dsX <- 'LiCN'
```

We repeat the parameter estimation on this data set.

```
if (short) {
  test.fpr <- c(0.15,0.185,0.22)
  test.fnr <- c(0.045,0.08,0.115)
} else {
  test.fpr <- test.fnr <- seq(from=0.01,to=0.5,length.out=15)
}

if (runParamEst) {
  dir.create(paste0('Res/',dsX),recursive = TRUE,showWarnings = FALSE)

  llh <- matrix(0,nrow = length(test.fpr), ncol=length(test.fnr))
  trees <- array(dim = c(length(test.fpr),length(test.fnr),ncol(D)))
  for (i.fnr in 1:length(test.fnr)) {
    for (i.fpr in 1:length(test.fpr)) {

      ## infer oncoNEM
      oNEM <- oncoNEM$new(Data=D,FPR=test.fpr[i.fpr],FNR=test.fnr[i.fnr])
      oNEM$search(delta=200)
      print(oNEM$TF$getBestCounter( ))

      llh[i.fpr,i.fnr] <- oNEM$best$llh
      trees[i.fpr,i.fnr,] <- oNEM$best$tree

      ## save output in every round in case something goes wrong
      save(llh,trees,file=paste0('Res/',dsX,'/llh_and_trees.RData'))
    }
  }
} else {
  load(paste0('Res/',dsX,'/llh_and_trees.RData'))
}

##-----------------------------------------------------------------------------
## Evaluate results of parameter estimation
```

```
## find best parameter combination
indx <- which(llh==max(llh),arr.ind=TRUE)
fpr.est <- test.fpr[indx[1]]
fnr.est <- test.fnr[indx[2]]
param.est <- data.frame(x=fpr.est,y=fnr.est,value="Parameter estimate")

save(param.est,fpr.est,fnr.est,test.fpr,test.fnr,indx,
     file=paste0('Res/',dsX,'/paramEst.RData'))
```

Again, we plot heatmaps of the likelihood landscape.

```
## ---------------------------------------------------------------------------
## Plot likelihood distribution for parameter combinations

load(paste0('Res/',dsX,'/llh_and_trees.RData'))
load(paste0('Res/',dsX,'/paramEst.RData'))

plotTitle <- 'Bladder cancer - LOH'

## format data for ggplot
df <- cbind(expand.grid(test.fpr,test.fnr),
            llh[as.matrix(expand.grid(1:length(test.fpr),
                                      1:length(test.fnr)))])
colnames(df) <- c('fpr','fnr','llh')
p <- ggplot(df, aes(fpr,fnr)) +
  geom_raster(aes(fill = llh),hjust = 0.5, vjust = 0.5)+
  geom_point(data=param.est,aes(x=x,y=y,shape=value),
             colour="black",bg="white",size=6) +
  scale_shape_manual(values = c('Parameter estimate'=25)) +
  labs(x = 'False positive rate',
       y = 'False negative rate') +
  scale_fill_gradientn(colours = rainbow(8)[1:7],
                       name='Log-likelihood',
                       limits=c(max(llh)-1000+5,max(llh)+5)) +
  theme(axis.text  = element_text(size=12),
        axis.title = element_text(size=16),
        axis.title.y = element_text(vjust = 1),
        strip.text.x = element_text(size=16),
        legend.text = element_text(size=16),
        legend.title = element_blank(),
        aspect.ratio = 1,
        plot.title = element_text(face="bold")) +
  ggtitle(plotTitle)

print(p)
```

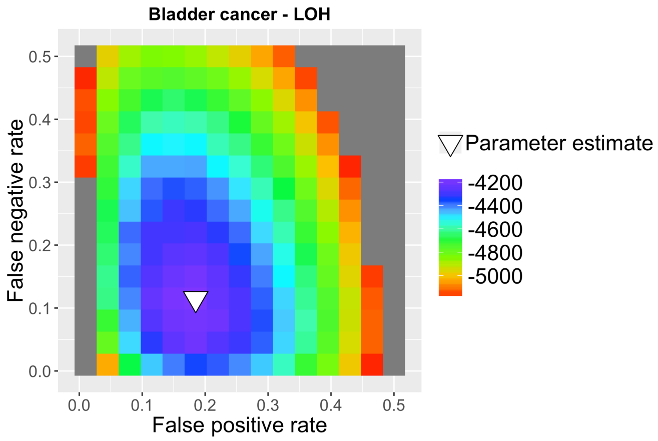

The estimated parameters for the LOH-blacklisted bladder cancer data set are FPR = 0.185 and FNR = 0.115. Using these parameters we infer the oncoNEM tree and plot it.

```
if (!file.exists(paste0('Res/',dsX,'/inferredTree_fpr-',fpr.est,'_fnr-',
                        fnr.est,'.RData'))) {
  load(paste0('Res/',dsX,'/llh_and_trees.RData'))
  load(paste0('Res/',dsX,'/paramEst.RData'))

  oNEM <- oncoNEM$new(Data=D,FPR=fpr.est,FNR=fnr.est)
  ## serach for unobserved nodes
  oNEM.expanded <- expandOncoNEM(oNEM,epsilon = 10,delta = 200,
                                 checkMax = 10000,app=TRUE)
  # cluster
  oncoTree <- clusterOncoNEM(oNEM = oNEM.expanded, epsilon=10)

  ssave(c('oNEM','oNEM.expanded','oncoTree'),
        file=paste0('Res/',dsX,'/inferredTree_fpr-',fpr.est,'_fnr-',
                    fnr.est,'.RData'))
}

load(paste0('Res/',dsX,'/inferredTree_fpr-',fpr.est,'_fnr-',
            fnr.est,'.RData'))
Data <- oNEM$Data
colnames(Data) <- 1:ncol(Data)
post <- oncoNEMposteriors(tree = oncoTree$g,
                          clones = oncoTree$clones,
                          Data = Data,
                          FPR = oNEM$FPR,FNR = oNEM$FNR)

par(mar=c(1,3,3,1))
plotTree(tree = oncoTree$g,clones = oncoTree$clones,
         e.length = colSums(post$p_theta)[-1],
         label.length = 20,axis=FALSE,edge.arrow.mode='-',
         vertex.color='white',
         v.label.cex=0.9)
```

```
## add customized y axis
axis(2, labels = seq(0,200,by=50), at = seq(0,-200,by=-50))

## add overall title
title(main=list("Bladder cancer - LOH",cex=1.5))
```

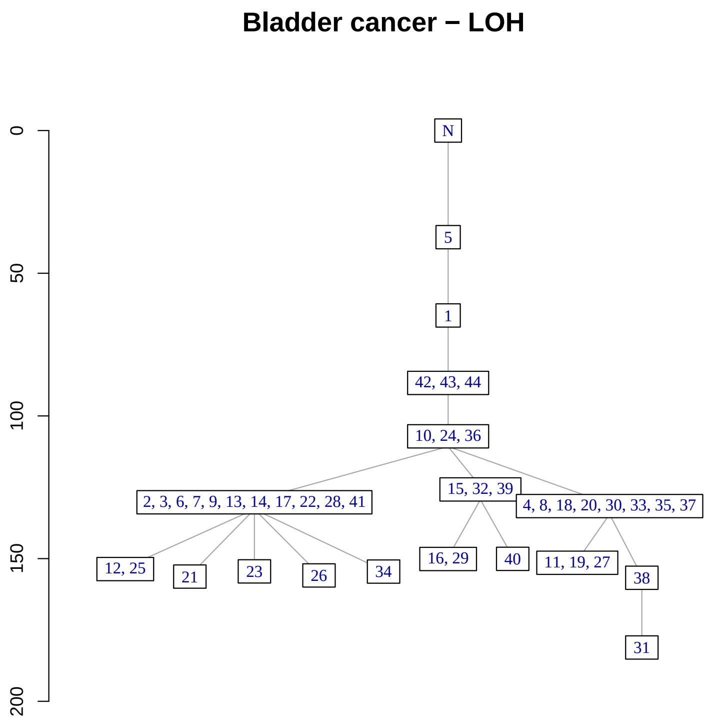

5 TCC — Comparison of oncoNEM results to results by Li et al

Li et al identified 8 genes in their data set that are recurrently mutated in bladder cancer. Based on their clustering and co-occurrence analysis, they inferred which of their 3 subpopulations are characterized by which of the 8 mutations.

In the next step we calculate the posterior probabilities of θ for those eight mutations and compare the results.

```
load('Res/Li/paramEst.RData')
load(paste0('Res/Li/inferredTree_fpr-',fpr.est,'_fnr-',fnr.est,'.RData'))

post <- oncoNEMposteriors(tree = oncoTree$g,
                          clones = oncoTree$clones,
                          Data = oNEM$Data,
                          FPR = oNEM$FPR,
                          FNR = oNEM$FNR)
postTheta.recurrGenes <- round(post$p_theta[Li.recurrGenes$gtyp.indx,],digits=2)

## manually order columns so that they are roughly grouped by larger branches in
## inferred tree and by temporal order
newLabels <- c(0,1,4,8,2,5,9,11,12,13,6,10,7,14,3)
postProb <- as.data.frame(postTheta.recurrGenes[,order(newLabels)])
newLabels[1] <- 'N'

## name rows and columns
colnames(postProb) <- c('N',1:14)
rownames(postProb) <- paste0(Li.recurrGenes$Gene.Name,'_',Li.recurrGenes$coord)
```

```
## assign clones to mutations as described by Li et al
postProb <- cbind(clone=c('A','C','A','A','A','C','C','B'),postProb)
## order rows by clone
postProb <- postProb[order(postProb$clone),]
## remove values for normal (always 0) to save space
postProb$N <- NULL

tab <- xtable(postProb, digits=2)
print(tab, size="\\scriptsize")
```

	clone	1	2	3	4	5	6	7	8	9	10	11	12	13	14
ASTN1_1,175400230,1,G/T	A	0.25	0.25	0.51	0.00	0.00	0.00	0.00	0.00	0.00	0.00	0.00	0.00	0.00	0.00
DHX57_2,38887207,1,G/A	A	0.45	0.45	0.09	0.00	0.00	0.00	0.00	0.00	0.00	0.00	0.00	0.00	0.00	0.00
NIPBL_5,37036306,1,G/A	A	0.33	0.33	0.33	0.00	0.00	0.00	0.00	0.00	0.00	0.00	0.00	0.00	0.00	0.00
CFTR_7,117054876,1,C/T	A	0.45	0.45	0.09	0.00	0.00	0.00	0.00	0.00	0.00	0.00	0.00	0.00	0.00	0.00
ATM_11,107611693,1,G/C	B	0.00	0.00	0.00	1.00	0.00	0.00	0.00	0.00	0.00	0.00	0.00	0.00	0.00	0.00
COL6A3_2,237914310,1,C/G	C	0.07	0.07	0.07	0.00	0.00	0.00	0.00	0.00	0.00	0.76	0.01	0.01	0.00	0.00
KIAA1958_9,114376732,1,C/T	C	0.00	0.00	0.00	0.00	0.00	0.00	0.00	0.00	0.00	0.98	0.00	0.00	0.00	0.00
KIAA1958_9,114376902,1,C/T	C	0.19	0.19	0.19	0.02	0.00	0.02	0.00	0.00	0.00	0.38	0.00	0.00	0.00	0.00

The clone labels in the table match the labels in the following two trees:

```
## plot schematic view of tree by Li et al
plotTree(tree=c(0,1,1),clones=c('N','A','B','C'))
title('Schematic representation of result by Li et al')
## plot Tree where nodes are labelled as in table
plotTree(tree=oncoTree$g,clones=newLabels,e.length=colSums(post$p_theta)[-1])
title('oncoNEM tree')
```

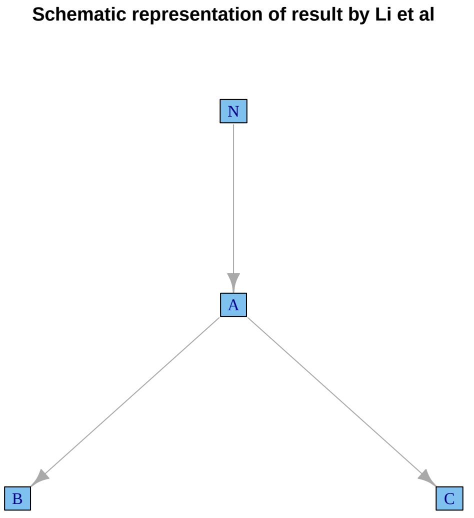

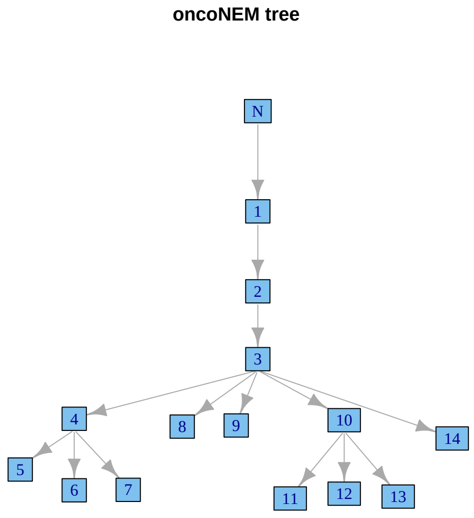

By comparing the clone positions and the mutation assignments between the two approaches, we can see that 7 out of 8 mutations are assigned to the same correspoding clones.

Finally, we plot the oncoNEM tree in which each cell carries the clone label assigned by Li et al, to assess if cells were assigned to corresponding clones by the two methods.

```
plotTree(tree = oncoTree$g,
         clones = lapply(oncoTree$clones,
```

```
                    function(x) Li.annot$cloneid[x]),
        e.length = colSums(post$p_theta)[-1],
        label.length = "max",axis=TRUE,edge.arrow.mode='-',
        vertex.color=c('paleturquoise3',
                    rep('thistle',igraph::vcount(oncoTree$g)-1)),v.label.cex=0.7)
```

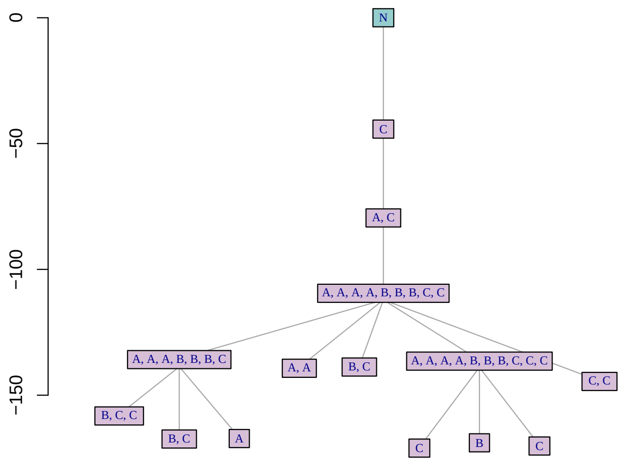

This shows that the assignment of cells to clones differs between the two approaches.

6 ET — Comparison of oncoNEM results to results obtained with BitPhylogeny by Yuan et al

For comparison we scored the tree inferred with BitPhylogeny by Yuan et al using the oncoNEM scoring model and based on the error rates we estimated.

```
load('Res/Hou/paramEst.RData')
load(paste0('Res/Hou/inferredTree_fpr-',fpr.est,'_fnr-',fnr.est,'.RData'))

## clone labels for every cell (obtained from Yuan et al, personal correspondence)
mapLabels <- c(0, 2, 10, 2, 11, 2, 2, 2, 2, 5, 2, 2, 2, 7, 4, 7, 2, 5,
               4, 7, 2, 8, 7, 1, 2, 2, 2, 2, 10, 2, 2, 2, 2, 1, 2, 2,
               1, 2, 2, 7, 2, 1, 8, 2, 7, 2, 2, 2, 1, 2, 1, 2, 5, 2, 2,
               5, 1, 10, 2, 2)

clones <- lapply(0:11,function(i) which(mapLabels[3:length(mapLabels)]==i))

## manually input BitPhylogeny tree
el <- rbind(c(0,1),
            c(1,2),
            c(0,3),
            c(3,4),
            c(3,5),
```

```
            c(0,6),
            c(6,7),
            c(6,8),
            c(0,9),
            c(9,10),
            c(0,11))+1 ## make 1-based
BitphyloScore <- scoreTree(igraph::graph.edgelist(el),clones = clones,
                           Data = Hou,FPR = fpr.est,FNR=fnr.est,
                           indx.inferred = TRUE)
```

Given the error rates inferred by oncoNEM, the log-likelihood of the BitPhylogeny tree is -11622, whereas the oncoNEM tree has a log-likelihood of -9964. This shows that the differences are not due to the heuristic nature of oncoNEM's search algorithm, but instead suggests that BitPhylogeny did not converge to the optimal solution.

7 Session Info

```
sessionInfo()

## R version 3.1.3 (2015-03-09)
## Platform: x86_64-apple-darwin10.8.0 (64-bit)
## Running under: OS X 10.8.5 (Mountain Lion)
##
## locale:
## [1] en_GB.UTF-8/en_GB.UTF-8/en_GB.UTF-8/C/en_GB.UTF-8/en_GB.UTF-8
##
## attached base packages:
## [1] stats     graphics  grDevices utils     datasets  methods   base
##
## other attached packages:
## [1] xtable_1.7-4   ggplot2_2.0.0 oncoNEM_1.0   Rcpp_0.11.6   knitr_1.11
##
## loaded via a namespace (and not attached):
##  [1] codetools_0.2-11 colorspace_1.2-6 digest_0.6.8     evaluate_0.8
##  [5] formatR_1.2      ggm_2.3          grid_3.1.3       gtable_0.1.2
##  [9] highr_0.5        igraph_0.7.1     labeling_0.3     magrittr_1.5
## [13] munsell_0.4.2    plyr_1.8.3       scales_0.3.0     stringi_0.4-1
## [17] stringr_1.0.0    tools_3.1.3
```

References

[1] Yingrui Li, Xun Xu, Luting Song, Yong Hou, Zesong Li, Shirley Tsang, Fuqiang Li, Kate Im, Kui Wu, Hanjie Wu, Xiaofei Ye, Guibo Li, Linlin Wang, Bo Zhang, Jie Liang, Wei Xie, Renhua Wu, Hui Jiang, Xiao Liu, Chang Yu, Hancheng Zheng, Min Jian, Liping Nie, Lei Wan, Min Shi, Xiaojuan Sun, Aifa Tang, Guangwu Guo, Yaoting Gui, Zhiming Cai, Jingxiang Li, Wen Wang, Zuhong Lu, Xiuqing Zhang, Lars Bolund, Karsten Kristiansen, Jian Wang, Huanming Yang, Michael Dean, and Jun Wang. Single-cell sequencing analysis characterizes common and cell-lineage-specific mutations in a muscle-invasive bladder cancer. *GigaScience*, 1(1):12, 2012.

[2] Yong Hou, Luting Song, Ping Zhu, Bo Zhang, Ye Tao, Xun Xu, Fuqiang Li, Kui Wu, Jie Liang, Di Shao, Hanjie Wu, Xiaofei Ye, Chen Ye, Renhua Wu, Min Jian, Yan Chen, Wei Xie, Ruren Zhang, Lei Chen, Xin Liu, Xiaotian Yao, Hancheng Zheng, Chang Yu, Qibin Li, Zhuolin Gong, Mao Mao, Xu Yang, Lin Yang, Jingxiang Li, Wen Wang, Zuhong Lu, Ning Gu, Goodman Laurie, Lars Bolund, Karsten Kristiansen, Jian Wang, Huanming Yang, Yingrui Li, Xiuqing Zhang, and Jun Wang. Single-cell exome sequencing and monoclonal evolution of a JAK2-negative myeloproliferative neoplasm. *Cell*, 148(5):873–885, Mar 2012.
